# IL-17 promotes osteoclast-induced bone loss by regulating glutamine-dependent energy metabolism

**DOI:** 10.1038/s41419-024-06475-2

**Published:** 2024-02-05

**Authors:** Renpeng Peng, Yimin Dong, Meng Zheng, Honglei Kang, Pengju Wang, Meipeng Zhu, Kehan Song, Wei Wu, Feng Li

**Affiliations:** Department of Orthopedic Surgery, Tongji Hospital, Huazhong University of Science and Technology, Wuhan, China

**Keywords:** Cell signalling, Transcription

## Abstract

Osteoclasts consume an amount of adenosine triphosphate (ATP) to perform their bone resorption function in the development of osteoporosis. However, the mechanism underlying osteoclast energy metabolism has not been fully elucidated. In addition to glucose, glutamine (Glu) is another major energy carrier to produce ATP. However, the role of Glu metabolism in osteoclasts and the related molecular mechanisms has been poorly elucidated. Here we show that Glu is required for osteoclast differentiation and function, and that Glu deprivation or pharmacological inhibition of Glu transporter ASCT2 by V9302 suppresses osteoclast differentiation and their bone resorptive function. In vivo treatment with V9302 improved OVX-induced bone loss. Mechanistically, RNA-seq combined with in vitro and in vivo experiments suggested that Glu mediates the role of IL-17 in promoting osteoclast differentiation and in regulating energy metabolism. In vivo IL-17 treatment exacerbated OVX-induced bone loss, and this effect requires the participation of Glu or its downstream metabolite α-KG. Taken together, this study revealed a previously unappreciated regulation of IL-17 on energy metabolism, and this regulation is Glu-dependent. Targeting the IL-17–Glu–energy metabolism axis may be a potential therapeutic strategy for the treatment of osteoporosis and other IL-17 related diseases.

## Introduction

Postmenopausal osteoporosis is the most common metabolic diseases of the skeleton [1], characterized by low bone mineral density and increased risk of fragile fractures, including hip and vertebral fractures, which result in substantial disability and socioeconomic burden worldwide [2, 3]. Due to the lack of estrogen, osteoclasts are overactivated in a postmenopausal condition, leading to excessive bone resorption and massive bone loss. Targeting the differentiation and function of osteoclasts is a main strategy for the treatment of osteoporosis.

The dynamitic process of bone remodeling, consisting of osteoblastic bone formation and osteoclastic bone resorption, is critical for maintaining bone mass [4]. Osteoclasts are multinucleated cells differentiating from bone marrow-derived macrophages (BMDMs) [5, 6]. During osteoclast differentiation, macrophage colony-stimulating factor (M-CSF) induces the formation of BMDMs, while the receptor activator of nuclear factor-κB ligand (RANKL) initiates the differentiation signaling to form osteoclasts. Overactivated osteoclasts are responsible for many pathological conditions, such as rheumatoid arthritis, Paget’s disease and osteoporosis [7]. During bone resorption, osteoclasts secret proteolytic enzymes and protons to dissolve organic and inorganic components of the bone matrix, a process fueled by energy from ATP [8, 9]. It has been reported that the differentiation and function of osteoclasts require metabolic adaptations, including increased oxidative phosphorylation and mitochondrial activity [5, 8, 10]. Targeting metabolic adaptations of osteoclasts during their differentiation may be a novel anti-resorptive therapeutic strategy. However, the molecular mechanisms underlying metabolic adaptation to the increased energy demands of osteoclasts remain largely unknown.

Both glucose and Glu are essential carbon sources that fuel the tricarboxylic acid cycle to support the high energy demands during osteoclast differentiation [11, 12]. Deprivation of Glu in the culture medium has been reported to suppress osteoclast differentiation and function [11]. However, the molecular mechanism underlying Glu mediated osteoclastogenesis is unclear. Extracellular Glu is transported into cells by ASCT2, a membrane transporter encoded by the *Slc1a5* gene, and then converted to glutamate in the mitochondria by a deamination reaction catalyzed by glutaminase (GLS1). Glutamate is converted by Glutamate dehydrogenase (GDH), Alanine aminotransferase (ALT) or Aspartate aminotransferase (AST) to the TCA cycle intermediate alpha-ketoglutaric acid (alpha-KG). In this study, we confirmed the role of Glu in promoting osteoclast differentiation. Besides, by using RNA-sequencing and bioinformatic analysis, we found that Glu is involved in the IL-17 signaling pathway to facilitate the promoting effects of IL-17 on osteoclast differentiation. Besides, the current study for the first time revealed a role of IL-17 in regulating energy metabolism, and this regulation depends on Glu. Targeting the IL-17–Glu–energy metabolism axis may be a novel therapeutic strategy for the treatment of osteoporosis and for other osteoclast-related diseases.

## Method and materials

### Reagents

V9302 was purchased from MedChemExpress (HY-W015229, MedChemExpress, USA). Recombinant RANKL (#462–TR), M-CSF (#416–ML) and IL-17 were purchased from R&D Systems (Minnesota, USA). Antibodies targeting CTSK (#ab37259) and ACP5 (#ab52750) were purchased from Abcam (Cambridge, UK). Antibodies against ASCT2 (#12782), NFATc1 (#8032), MMP9 (#3852), P38 (#8690), JNK (#9252), ERK (#), P65 (#8242), IkBa (#4812), p-P38 (#4511), p-JNK (#9255), p-ERK (#4370), p-P65 (#3033), p-IkBa (#2859) and TRAF6 were obtained from Cell Signaling Technology (Beverly, MA, USA). Antibodies against TRAF3IP2 (#A6776), CXCL10(#A1457), MMP3(#A1202), MMP13(#A1606), C/EBPB (#A19538) were obtained from ABclonal (Wuhan, China). a-modified essential medium (a-MEM) was obtained from Boster (Boster Bio, Pleasanton, USA), a-MEM without L-Glu from Boster (Boster Bio, Pleasanton, USA). L-Glu was purchased from Sigma (Sigma, United States). Glu replete medium was made by adding 0.5/1.0/2.0/4.0 mM L-Glu to the Glu deprivation medium. Dimethyl-a-ketoglutarate (DM-α-KG) was purchased from Sigma (Sigma, United States)

### BMM isolation and in vitro OC differentiation assay

Osteoclast differentiation was induced as previously reported [9, 13]. Briefly, BMDMs were extracted from the medullary space of tibias and femurs of 8-week-old C57BL/6 J mice and were cultured in α-MEM medium with 30 ng/ml M-CSF and 10% fetal bovine serum (FBS). After 14 h, suspension cells were collected and transferred to new 10-cm dishes, followed by culture in α-MEM medium with 30 ng/ml M-CSF for additional three days. The adherent cells were then washed and digested with 0.25% trypsin for further use. For osteoclast differentiation assay, cells were seeded on 96-well plates at a density of 2 × 10^4^ per well. After cell adhesion, the medium was replaced with fresh α-MEM containing 30 ng/ml M-CSF and 75 ng/ml RANKL to induce osteoclast differentiation.

### TRAP staining, F-Actin ring formation, and pit formation assay

TRAP staining kit (387A-1KT, Sigma, United States) was used to stain osteoclasts after differentiation. Briefly, the cells were washed with PBS for three times and were fixed with 4% paraformaldehyde for 10 min, followed by washing with ddH_2_O for three times. Then, the staining buffer were prepared according to the manufacturer’s instruction. The fixed cells were incubated with the staining buffer at 37 °C for 1 h, and the whole wells were scanned under a scanning microscope. TRAP-positive multinucleated cells with three or more nuclei were counted as osteoclasts.

F-actin ring formation was analyzed by staining with rhodamine-conjugated phalloidin (Invitrogen, Carlsbad, CA, USA). BMDMs were seeded onto hydroxyapatite-coated Osteo Assay strip well plates (#3988, Corning, Inc, USA), and were treated with different concentrations of Glu in osteoclast-inducing medium. After five days of treatment, the cells were fixed with 4% paraformaldehyde (Sigma-Aldrich, Shanghai, China) and 0.1% Triton X-100 was applied for permeabilization. After being washed for three times, the cells were stained with rhodamine-conjugated phalloidin in 2% bovine serum albumin (1: 200) for 1 h. Nuclei were visualized by 40, 6-diamidino-2-phenylindole (DAPI) staining for 5 min. Fluorescence images of F-actin were acquired using a fluorescence microscope (Leica, Munich, Germany). Three fields of each well were randomly captured, and ImageJ software (NIH) was used to quantify the number of F-actin rings. For pit formation assay, the cells were digested with trypsin and washed off with running water to expose the surface of the well bottom. The osteoclastic bone resorption pits were captured under light microscopy. Image J software (National Institutes of Health) was used to measure the resorption area.

### Real-time quantitative PCR (RT-qPCR)

The total mRNA of cultured cells was extracted using TRIzol reagent (Takara, Japan). A total of 1 μg mRNA was reverse transcribed into cDNA by using HiScript III All-in-one RT SuperMix Perfect for qPCR (Vazyme Biotech Co., Ltd, Nanjing, China) and real-time PCR analyses were performed using the CFX connect Real-Time System with HiScript II Q RT SuperMix for qPCR (Vazyme Biotech Co.,Ltd, Nanjing, China). All the mRNA levels of the target genes were normalized to the level of housekeeping gene β-Actin. The primer sequences used were listed in supplementary table S1.

### Western blot assay

The treated cells were washed with pre-chilled PBS and lysed with RIPA buffer containing phosphotransferase inhibitor (1%) and proteinase inhibitor (1%). The concentration of total protein in the lysis buffer was measured with the BCA kit (BOSTER, Wuhan, China). For Western blot, a total of 20 μg protein was separated on 10% SDS–PAGE by electrophoresis (120 V, 60 min) and was transferred to a polyvinylidene difluoride (PVDF) membrane (275 mA, 80 min). The PVDF membranes were then blocked with 5% BSA solution at room temperature for 1 h, followed by incubation with the primary antibodies at 4 °C overnight. Horseradish peroxidase-conjugated anti-mouse or anti-rabbit IgG was used as secondary antibodies. Chemiluminescence (Yeasen, Super ECL Detection Reagent, 36208ES60) was used to detect the protein signals.

### RNA sequencing and data analysis

RNA sequencing was performed by Novogene (Beijing, China). BMDMs were divided into Glu deprivation group and control group, with 3 replicates in each group. The total RNA was extracted with RNAeasy Kit (Qiagen). Then, a total of 5 μg of RNA per sample was used as input material for RNA sample preparations, with mRNA purified from total RNA using poly-T oligo-attached magnetic beads. Sequencing libraries were constructed by using the NEBNext® UltraTM RNA Library Prep Kit for Illumina® (NEB, USA). Sequencing reads were aligned to the mouse reference genome mm10 (GRCm38.90) using STAR aligner (v2.5.1b) guided by the mouse GENCODE gene model release v15. HTSeq v0.6.0 was used to count the read numbers mapped to each gene. Then, the FPKM of each gene was calculated based on the length of the gene and the read count mapped to this gene. For data analysis, the “Limma” package in the R software was used to identify differentially expressed genes (DEG) between Glu deprivation group and the control group. DEGs were defined as those with at least one-fold change of expression and with an adjusted *P* value less than 0.05. KEGG and GSEA pathway analysis on the sequencing data was performed with the clusterProfiler package of R.

### Mouse model of ovariectomy (OVX) induced osteoporosis and in vivo treatment

The animal experiments were conducted according to the principles of the Ethics Committee of Tongji Hospital, Huazhong University of Science and Technology for the care and use of experimental animals. C57BL/6 female mice were housed in specific-pathogen-free rooms and OVX were performed at 12 weeks of age. Mice were randomly assigned into each group (*n* = 6). As previously reported, the OVX model was constructed by removing bilateral ovaries via a dorsal approach [13]. The sham surgery was performed via the same approach until exposing the bilateral ovaries. One week after surgery, the mice were intraperitoneally injected with V9302 or vehicles 4 days a week for six weeks.

### Micro-computed tomography (μCT)

The mice were sacrificed after six weeks of treatment, and the bilateral femurs were collected for μCT scanning or for histological staining. The parameters for mouse femur scanning were set to 100 kV, 98 μA, and 10 µm voxel size. The region from 0.05 mm below the growth plate to 5% of femoral length was selected for 3-dimensional histomorphometric analysis to determine cortical or trabecular bone mineral density (BMD). Trabecular bone morphometric parameters, including BV/TV (bone volume/tissue volume), Tb.N (trabecular number), Tb.Sp (trabecular separation/spacing), and Tb.Th (trabecular separation/trabecular thickness), were used to analysis the changes of the bone mass. The trabecular bone volume was visualized by three-dimensional reconstruction using the build-in software in the μCT system.

### Histo-morphometric analysis

Femur samples were fixed in 4% paraformaldehyde for 48 h, followed by decalcification with 15% ethylenediaminetetraacetic acid (EDTA) for 2 weeks. The femur samples were then embedded in paraffin and were then sectioned into slices (6 μm) for TRAP and HE staining. TRAP staining was used to visualize osteoclast formation in femur bone tissues, whereas toluidine blue plus fast green staining was used to show osteoblasts. Image J software was used to calculate osteoclast/bone surface (Oc.S/BS (%)) and number of osteoblasts per bone perimeter (N.Ob/B.Pm).

### Extracellular acidification rate (ECAR) and oxygen consumption rate (OCR)

A Seahorse XF24 Extracellular Flux Analyser (Agilent Technologies, CA, USA) was used to measure the OCR and ECAR. Briefly, stimulated cells were seeded in XF24 plates at a density of 3 × 10^4^ cells per well, and the plates were detected according to the instructions of the XFp Cell Mito Stress Test Kit (103015-100), XFp Glycolysis Stress Test Kit (103020-100) or XFp Mito Fuel Flex Test Kit (103270-100). Data were assessed with the XFe Wave Software. All data were normalized to the cell number.

### Detection of lactate concentration in the culture media

The Lactate Assay kit (Beyotime Biotechnology, China) was used to detect the concentration of lactate in the culture media. Briefly, cells were cultured in 6-well plates at a density of 1 × 10^6^ cells per well. Osteoclast precursors were cultured with different interventions for three days. Then, the culture medium was collected and was subjected to the lactate detecting assay. All procedures were performed according to the manufacture’s instruction.

### Statistical analysis

All data representative of three independent experiments are present as mean ± SEM. We used two-tailed t tests to determine the statistical significance between two groups. We performed multiple group comparisons by one- or two-way ANOVA analysis with Bonferroni post-test in GraphPad prism version 5. For all statistical tests, we considered *P* value < 0.05 to be statistically significant.

## Results

### Glu is required for osteoclast differentiation

**Fig. 1:**
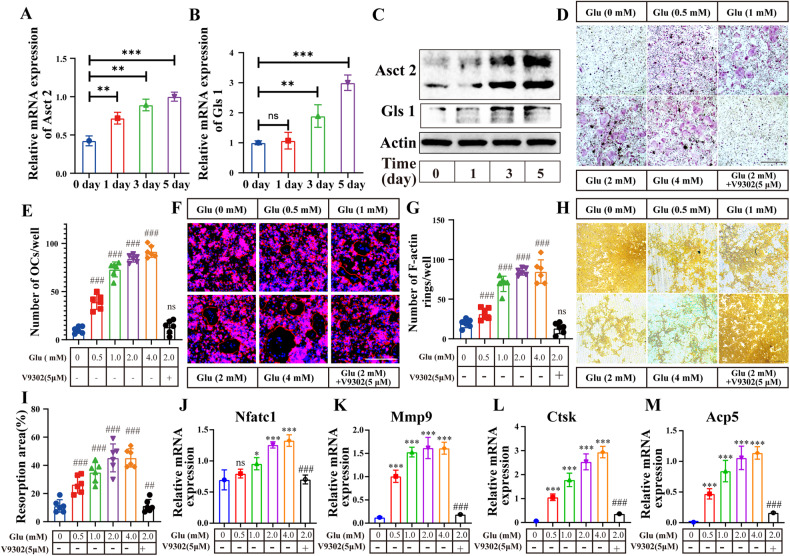
Glu is required for osteoclast differentiation. **A**–**C** The expression of Asct2 and Gls1 at the mRNA and protein level on day 1, 3, and 5 during OC differentiation. Statistical significance: *p < 0.05, **p < 0.01, ***p < 0.001; ns, no significance. **D**, **E** Glu regulate RANKL-induced osteoclastogenesis in a concentration-dependent manner. BMDMs were treated with different concentrations of Glu (0 mM,0.5 mM, 1 mM, 2 mM and 4 mM) or 5 μM V9302 for 5 days. TRAP-positive multinucleated (>3 nuclei) cells were counted as osteoclasts. *p < 0.05, **p < 0.01, ***p < 0.001. **F**, **G** BMDMs were seeded on 0.2% collagen-gel-coated 6-well plates and stimulated with 30 ng/ml M- CSF and 75 ng/ml RANKL for 6 days. Then, the cells were digested and seeded onto the Osteo Assay stripwell plates. Mature osteoclasts were treated with various concentrations of Glu or 5 μM V9302 for 5 days. F-actin staining was then performed. *p < 0.05, **p < 0.01, ***p < 0.001. **H**, **I** Pit formation assay in the Glu (0 mM,0.5 mM, 1 mM, 2 mM and 4 mM) or 5 μM V9302 treated group. Mature osteoclasts were cultured on Osteo Assay stripwell plates and treated with the medium containing RANKL and various concentrations of Glu for 2 days. The cells were then washed from the surface by using the 10% bleaching solution for 5 min. Resorption pits were captured with light microscopy and analyzed with Image J software. *p < 0.05, **p < 0.01, ***p < 0.001. **J**–**M** BMDMs were cultured with the medium containing M- CSF, RANKL and various concentrations of Glu or were treated by 5 μM V9302 for 5 days. Relative mRNA expression levels of Nfatc1, Mmp9, Ctsk, and Acp5 versus β-Actin were quantified by qPCR. *compared to Glu (0 mM)/V9302 (0 μM) group; #compare to Glu (2 mM)/V9302(0 μM) group; *p < 0.05, **p < 0.01, ***p < 0.001. #*p* < 0.05, ##*p* < 0.01, ###*p* < 0.001.

It has been reported that Glu plays an essential role in the process of osteoclast differentiation [11]. Glu is one of the major energy substrates of cell metabolism, we first examined the proliferation rates of BMDM under different Glu concentrations and V9302 (5 μM) (a specific ASCT2 inhibitor that blocks Glu uptake) stimulation. BMDM proliferation was decreased in the absence of Glu, but were not changed in medium with 1–4 mM of Glu (Fig. S1A). During osteoclastogenesis, the expression of ASCT2 and GLS1 was significantly upregulated after RANKL treatment, at both mRNA (Fig. 1A, B) and protein levels (Fig. 1C, Fig. S1B, C), suggesting increasing demand of Glu by BMDMs and osteoclasts during differentiation. Sunsequently, we examined the expression of GLS1 and ASCT2 under different Glu concentrations and V9302 (5 μM) (a specific ASCT2 inhibitor that blocks Glu uptake) stimulation. We found that both Glu deprivation and Glu supplementation had little effect on ASCT2 expression (Fig. S1D), whereas GLS1 expression was increased in Glu-deprived medium (Fig. S1E). We then induced osteoclast differentiation in conditioned Glu-deprived medium or in normal culture medium containing 5 μM V9302 [11]. Compared to the normal culture medium, the formation of osteoclasts and F-actin rings was significantly suppressed in the Glu-free or V9302-treated group (Fig. 1D–G). Pit formation assay showed that the resorption area in the Glu-depleted group or in the V9302 group was smaller than that in the control group (Fig. 1H, I). Osteoclast related markers, including Acp5, Mmp9, Ctsk and Nfatc1, were also significantly inhibited in the Glu-free medium or by V9302 treatment (Fig. 1J–M and Fig. S2A–E). Osteoclast differentiation, F-actin ring formation, osteoclast resorption activity, and the expression of osteoclast markers were partially reversed by supplementation of Glu to the Glu-free medium at a concentration of 0.25-fold (0.5 mM) and 0.5-fold (1 mM) of normal concentration, and were enhanced by 2-fold (4 mM) concentration of Glu in the medium. Alizarine red staining and ALP staining showed that the differentiation of osteoblasts was inhibited by complete Glu deprivation, but such inhibition was abrogated by even low Glu concentrations. Meanwhile, V9302 (5 μM) have no influence on osteoblast differentiation (Fig. S2F, H). Correspondingly, the expression of osteoblast related markers, including *Alpl*, *Opn*, *Ocn*, *Col1a1* and *Runx2*, was not changed by decreasing Glu concentration or by V9302 treatment, and was only suppressed in Glu deprived medium (Fig. S2I, M). These resulted suggest that osteoclasts are more sensitive to the changes of Glu concentration and are likely to be inhibited by shortage of Glu, while osteoblast differentiation was only affected by Glu deprivation.

### Blocking Glu uptake by V9302 attenuates osteoclast-induced bone loss in OVX mice

**Fig. 2:**
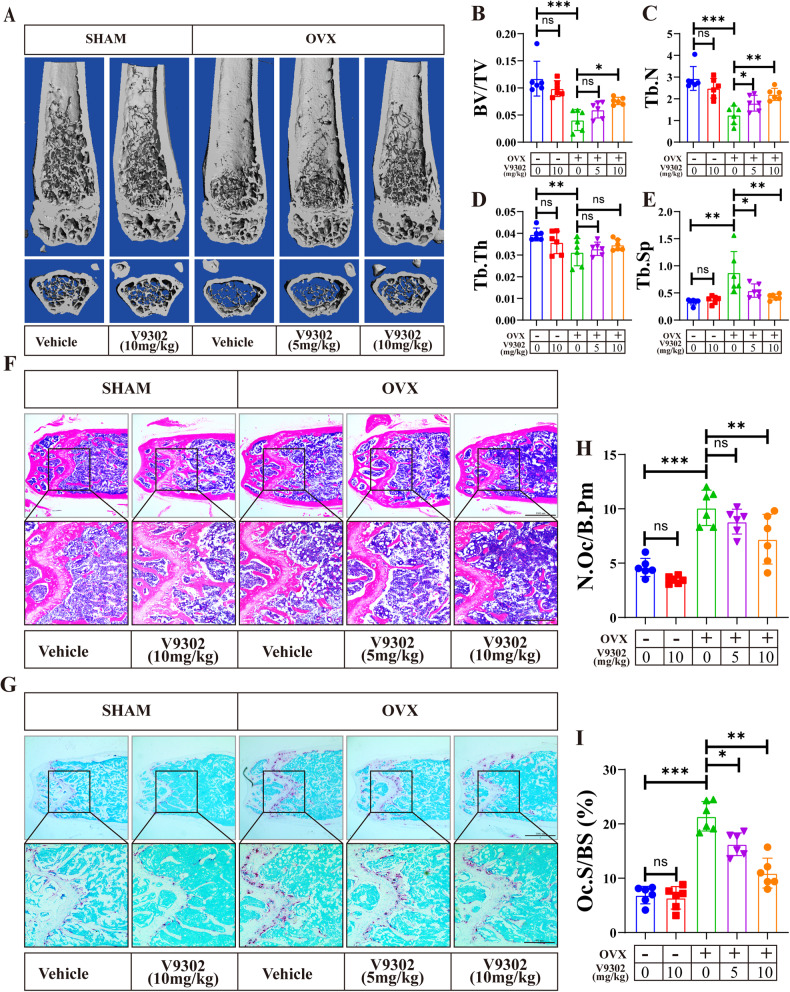
Blocking Glu uptake by V9302 attenuates osteoclast-induced bone loss in OVX mice. **A** Representative 3D-constructed images of the distal femurs of mice in each group. **B**–**E** Quantitative analyses of bone structural parameters of the distal femurs, including bone volume/tissue volume (BV/TV), Tb.N (trabecular number), trabecular thickness (Tb.Th), and trabecular space (Tb.Sp). **F** Representative sections of the distal femurs were performed with H&E staining. **G** Representative sections of the distal femurs were performed with TRAP staining. **H**, **I** Quantitative analyses of histomorphometric bone parameters, including N.Oc/B.Pm and Oc.S/BS were performed, to reflect the formation of mature OCs on bone tissue slices. All data are presented as the mean ± SEM. *p < 0.05, **p < 0.01, ***p < 0.001. *N* = 6–8 per group.

In the absence of estrogen, osteoclasts are overactivated and have increased resorptive function, which contribute to bone loss in postmenopausal women and in OVX mice. As Glu is required for osteoclast differentiation and for the resorptive function, we then asked whether blocking osteoclast Glu uptake can attenuate bone loss in OVX mice. We treated OVX mice with either V9302 (5 or 10 mg/kg/d) or vehicle for 8 weeks and collected the femurs of each mouse for bone morphologic and histologic analyses. Compared to the sham+vehicle group, V9302 (10 mg/kg) has no effects on bone mass in the sham group, and mice in the OVX+vehicle group showed significantly lower BV/TV, Tb.N, Tb.Th, BMC/TV, but higher T b.Sp (Fig. 2A–E, Fig. S3A, B), suggesting decreased bone mass due to OVX. However, treatment with low and high doses of V9302 (5 mg/kg and 10 mg/kg) significantly reversed the changes of these parameters (Fig. 2A–E, Fig. S3A, B), and the effects were dose-dependent. However, cortical bone parameters, including Ct. Ar and Ct. Th compared with the sham group, was not affected by V9302 treatment (Fig. S3C–E). HE staining and TRAP staining on the femur bone slices showed increased trabecular bone and less TRAP-positive cells within bone slices in the V9302-treated group (Fig. 2F, G). Besides, N.OC/B.Pm and OC. S/BS were also lower in the low- and high-dose V9302-treated groups (Fig. 2H, I) than that in the OVX group. Histomorphometric analysis of N.Ob/B.Pm or Ob.S/BS revealed no effects of V9302 on osteoblast differentiation (Fig. S3F–H). These results suggest that blocking ASCT2 mediated Glu uptake with V9302 can improve the osteoclast-induced bone loss in OVX mice.

### Glu deficiency leads to impaired energy metabolism in osteoclasts, which can be rescured by α-ketoGlutarate (α-KG)

**Fig. 3:**
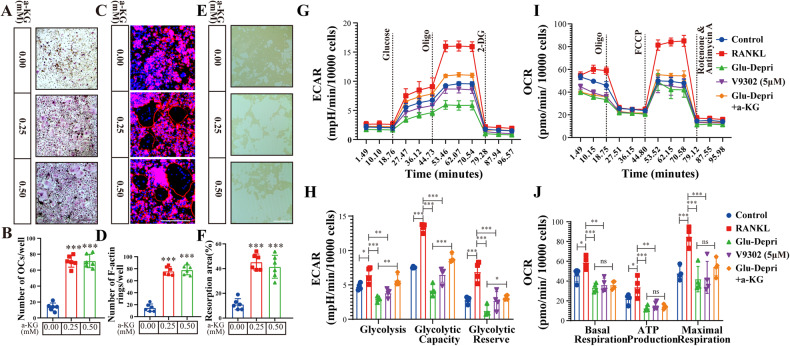
Glu deficiency leads to impaired energy metabolism in osteoclasts, which can be rescured by α-ketoGlutarate (α-KG). **A**, **B** BMDMs were cultured with the Glu deprived medium that containing M-CSF (30 ng/ml) and RANKL (75 ng/mL) for 5 days, as well as treated with indicated concentration of α-KG. TRAP-positive multinucleated (>3 nuclei) cells were counted as osteoclasts. **C**, **D** BMDMs were seeded on Osteo Assay stripwell plates and stimulated Glu deprived medium that containing M-CSF (30 ng/ml), RANKL (75 ng/mL) and indicated concentration of α-KG for 5 days. F-actin staining was then performed. **E**, **F** BMDMs were seeded on Osteo Assay stripwell plates and stimulated Glu deprived medium that containing M-CSF (30 ng/ml), RANKL (75 ng/mL) and indicated concentration of α-KG for 5 days. The cells were then washed from the surface by using the 10% bleaching solution for 5 min. Resorption pits were captured with light microscopy and analyzed with Image J software. **G**, **H** BMDMs seeded in Seahorse XF analyzer culture plates and treated as described in (**A**, **B**) Extracellular acidification rate (ECAR) were analyzed by XF Cell Mito Stress Assay. **I**, **J** BMDMs were seeded in Seahorse XF analyzer culture plates and treated as described in (**A**, **B**), Oxygen consumption rate (OCR) were analyzed by XF Cell Mito Stress Assay.

Glu is an important energy source to drive cellular physiological function. It is converted into α-KG and enters the TCA cycle for mitochondrial energy production [14, 15]. In Glu-free medium, osteoclast inhibition was reversed by the supplementation of α-KG (Fig. 3A, B). F-actin formation and bone resorptive function were also reversed by α-KG supplementation to the Glu-free medium (Fig. 3C–F). These results suggest that the deprivation of Glu may suppress osteoclast differentiation by reducing α-KG formation and affect energy metabolism in the TCA cycle. To further confirm this hypothesis, we measured the oxygen consumption rate (OCR) and extracellular acidification rate (ECAR) to assess mitochondrial respiratory function and glycolytic capacity of BMDMs after osteoclast induction. As shown in Fig. 3G–J, The OCR and ECAR of BMDMs were significantly increased after RANKL treatment at all time points, but was decreased by Glu-deprivation or by V9302 treatment. Supplementation of α-KG partially reversed the decrease of OCR and ECAR of BMDMs in the Glu-deprivation group (Fig. 3G–J). In addition, the lactate concentration in the medium of the RANKL treated group was higher than in the control group, whereas Glu-deprivation or V9302 treatment decreased lactate production after RANKL treatment (Fig. S4). These results suggest that Glu contributes to energy metabolism in osteoclasts by forming α-KG to provide fuels for the TCA cycle.

### Glu is involved in the IL-17 signaling pathway during osteoclast differentiation

**Fig. 4:**
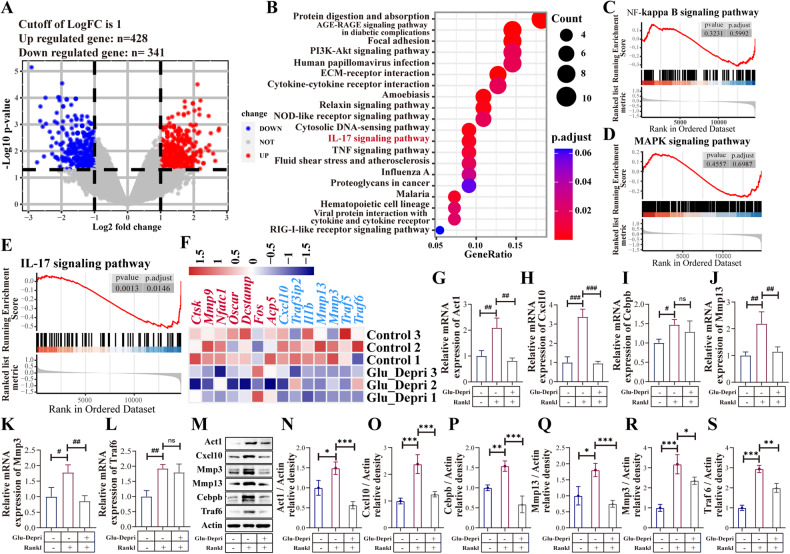
Glu is involved in the IL-17 signaling pathway during osteoclast differentiation. **A** Volcano plot showing the differentially expressed genes between the control group and Glu deprivation group. Differentially expressed genes were identified by setting the threshold of |log2 (fold change)| to 1 and the *P* value to 0.05. **B** KEGG enrichment analysis showed the IL-17 signaling pathway were significantly altered after Glu deprivation. **C**, **D** GSEA analysis confirmed that Glu deprivation have no influence on MAPK and NFκB pathways. **E** GSEA analysis confirmed the inhibition of IL-17 signaling pathway by Glu deprivation. **F** Heatmap showing the downregulated genes of osteoclast markers and genes in the IL-17 signaling pathway. **G**–**S** QPCR and Western blot assay examining the the expression of genes of osteoclast markers and genes in the IL-17 signaling pathway. *p < 0.05, **p < 0.01, ***p < 0.001; ns, no significance.

To investigate how Glu regulates osteoclast differentiation, we performed RNA-sequencing on BMDMs cultured in Glu-deprived medium and in the control medium after RANKL treatment. Using |log_2_FC | ≥1 and *p* < 0.05 as the criteria to screen for DEGs, we identified 428 upregulated and 341 downregulated DEGs in the Glu deprivation group **(**Fig. 4A**)**. After RANKL stimulation, the MAPK and NFκB pathways are the major activated pathways to initiate osteoclast differentiation [16]. However, the two pathways were not enriched in the KEGG analysis of DEGs between the two groups (Fig. 4B). GSEA analysis (Fig. 4C, D) showed that the two pathways were not significantly changed after Glu-deprivation. Interestingly, we found the IL-17 signaling pathway, which has been shown to facilitate osteoclast formation [17, 18], was significantly changed in the Glu-deprivation group (Fig. 4B, E). The heatmap also showed that the expression of genes in the IL-17 signaling pathway, along with osteoclast marker genes, were downregulated in the Glu deprivation group (Fig. 4F). To further confirm the RNA-sequencing results, we examined the expression of major genes in the IL-17 pathway, including Act1, Cxcl10, Mmp3, Mmp13, Traf6 and Cebpb, at the mRNA and protein level. We found RNAKL treatment significantly induced the expression of all of these genes, while Glu-deprivation decreased the expression of Act1, Cxcl10, Cebpb, Mmp3, Mmp13 and Traf6 (Fig. 4G–S). These results indicate that Glu is involved in the IL-17 signaling pathway during osteoclast differentiation.

### IL-17 depends on Glu to promote osteoclast differentiation

**Fig. 5:**
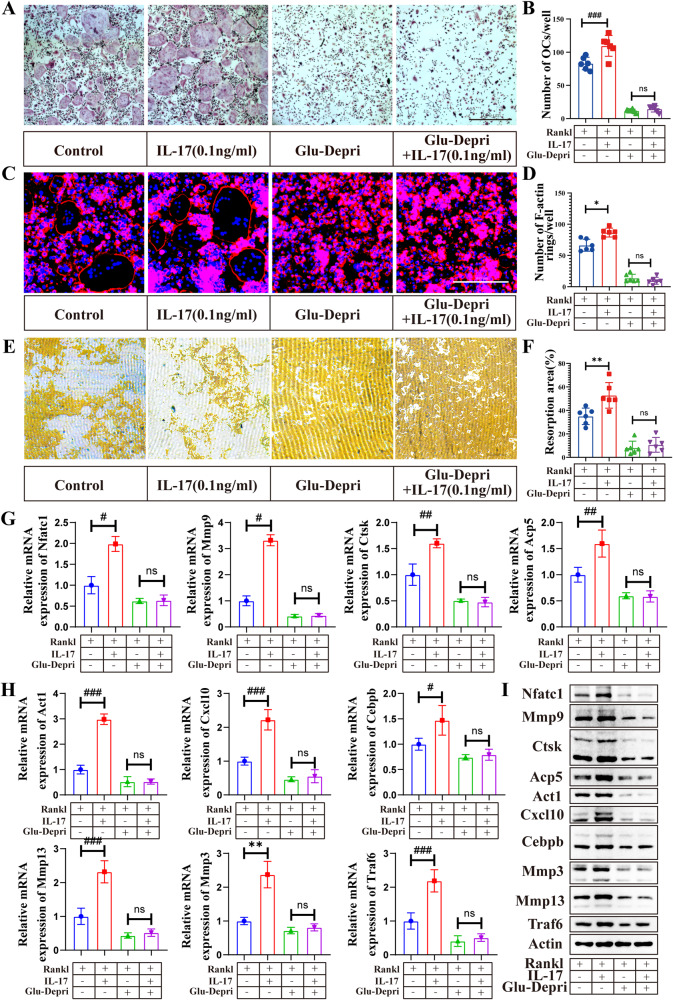
IL17 promotes osteoclast differentiation dependent on Glu. BMDMs were cultured with the medium that containing M-CSF (30 ng/ml) and RANKL (75 ng/mL) for 5 days with or without Glu deprivation, as well as treated with IL-17 (0.1 ng/ml). **A**, **B** TRAP-positive multinucleated (>3 nuclei) cells were counted as osteoclasts. **C**, **D** F-actin staining was then performed. **E**, **F** BMDMs were seeded on Osteo Assay stripwell plates and cultured with the medium that containing M-CSF (30 ng/ml) and RANKL (75 ng/mL) for 5 days with or without Glu deprivation, as well as treated with IL-17(0.1 ng/ml). The cells were then washed from the surface by using the 10% bleaching solution for 5 min. Resorption pits were captured with light microscopy and analyzed with Image J software. **G**, **H**, **I** BMDMs were seeded in 6 well plates and treated as described. QPCR and Western blot assay examining the the expression of genes of osteoclast markers and genes in the IL-17 signaling pathway. *p < 0.05, **p < 0.01, ***p < 0.001; ns, no significance.

Our previous study has reported that the IL-17 facilitates osteoclast differentiation [19]. However, the mechanism remains unclear. As Glu is required for the IL-17 signaling, we then investigated whether the effect of IL-17 on osteoclast differentiation is Glu-dependent. IL-17 at a concentration of 0.1 ng/ml significantly promoted osteoclast differentiation, F-actin ring formation (Fig. 5A–D) and the resorptive function (Fig. 5E, F). However, these promoting effects of IL-17 were abolished by Glu deprivation (Fig. 5A–F). IL-17 treatment increased the mRNA and protein expression of osteoclast marker genes, such as Nfatc1, Mmp9, Acp5, Ctsk, as well as genes involved in the IL-17 signaling pathway, such as Act1, Cxcl10, Mmp3, Mmp13, Cebpb and Traf6 (Fig. 5G–I, Fig. S5A–J). However, such increase was also prevented in the Glu-deprivation group after IL-17 treatment. These results suggest that the osteoclast-promoting role of IL-17 requires the presence of Glu.

### IL-17 regulates osteoclast energy metabolism via Glu

**Fig. 6:**
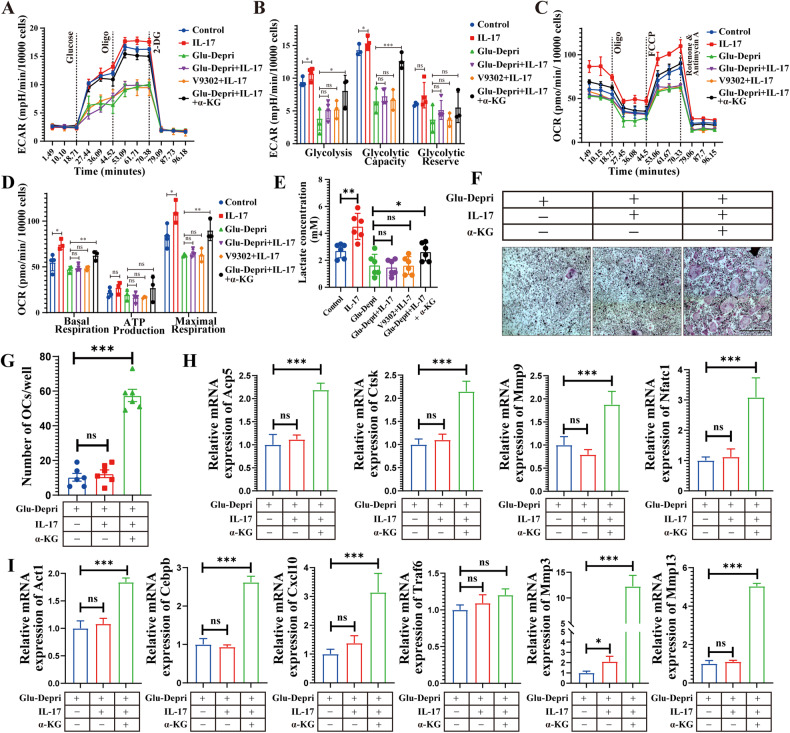
IL-17 regulates osteoclast energy metabolism via Glu. BMDMs were cultured with the medium that containing M-CSF (30 ng/ml) and RANKL (75 ng/mL) for 5 days with or without Glu deprivation, as well as treated with indicated stimulation, including IL-17(0.1 ng/ml), V9302 (5 μM) and α-KG (0.5 mM). **A**, **B** BMDMs were seeded in Seahorse XF analyzer culture plates and treated as described. Extracellular acidification rate (ECAR) was analyzed by XF Cell Mito Stress Assay. **C**, **D** BMDMs were seeded in Seahorse XFp analyzer culture plates and treated as described. Oxygen consumption rate (OCR) were analyzed by XF Cell Mito Stress Assay. **E** BMDMs were seeded in 6 well plates and treated as described. The levels of lactate were analyzed through Lactate Assay kit. **F**, **G** BMDMs were cultured with the Glu deprived medium that containing M-CSF (30 ng/ml) and RANKL (75 ng/mL) for 5 days, as well as treated with or without IL-17 (0.1 ng/ml) and α-KG (0.5 mM). TRAP-positive multinucleated (>3 nuclei) cells were counted as osteoclasts. **H**, **I** QPCR and Western blot assay examining the the expression of gene of osteoclast marker and IL-17 signaling pathway. *p < 0.05, **p < 0.01, ***p < 0.001; ns, no significance.

Since Glu is involved in osteoclast energy metabolism, we questioned whether the energy metabolism is controlled by IL-17. During osteoclast differentiation, the ECAR (Fig. 6A, B) and OCR (Fig. 6C, D) of osteoclasts were increased by IL-17 stimulation. IL-17 also increased lactate production by BMDMs (Fig. 6E). Interestingly, ECAR, OCR, and lactate production after IL-17 stimulation were all decreased by the deprivation of Glu. Supplementing α-KG to the medium partially rescued the decrease of ECAR, OCR, and lactate production due to Glu deprivation. Besides, IL-17 was able to promote osteoclast formation (Fig. 6F, G) and increase the expression of osteoclast marker genes when co-administered with α-KG to the Glu-deprivation medium (Fig. 6H). The expression of IL-17 signaling pathway genes was also increased by IL-17 and α-KG co-treatment in the Glu-deprivation medium group (Fig. 6I). These results indicate that IL-17 controls Glu/α-KG**–**dependent energy metabolism to promote osteoclast differentiation.

### The IL-17–Glu axis increases bone loss in OVX mice

**Fig. 7:**
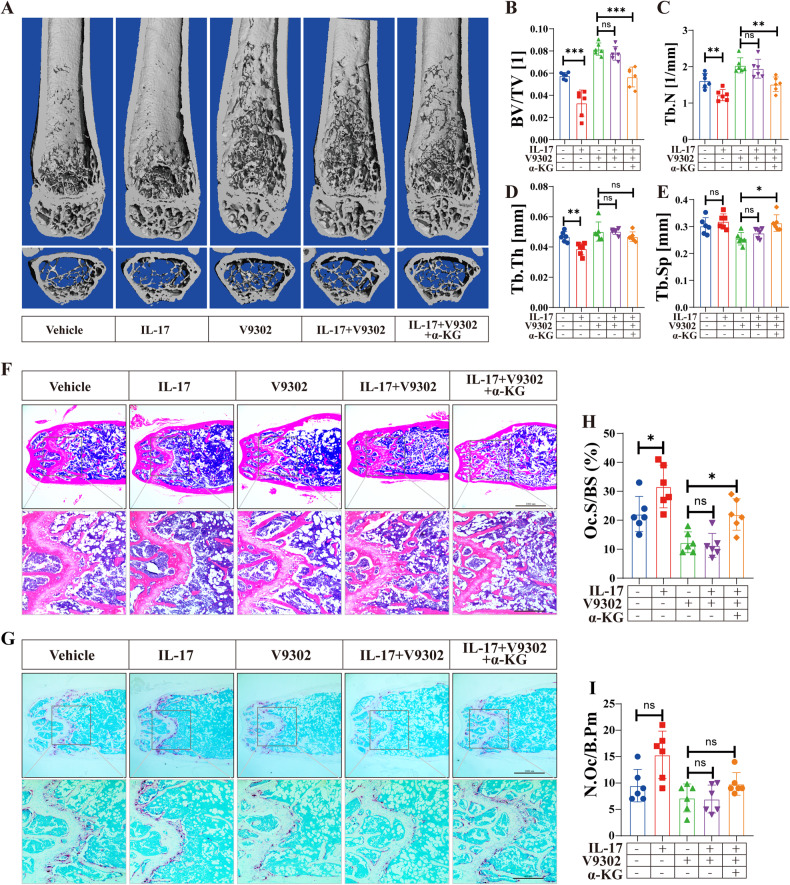
The IL17-Glu axis increases bone loss in OVX mice. **A** Representative 3D-constructed images of the distal femurs of mice in each group. **B**–**E** Quantitative analyses of bone structural parameters of the distal femurs, including bone volume/tissue volume (BV/TV), Tb.N (trabecular number), trabecular thickness (Tb.Th), and trabecular space (Tb.Sp). **F** Representative sections of the distal femurs were performed with H&E staining. **G** Representative sections of the distal femurs were performed with TRAP staining. **H**, **I** Quantitative analyses of histomorphometric bone parameters, including N.Oc/B.Pm and Oc.S/BS were performed, to reflect the formation of mature OCs on bone tissue slices. All data are presented as the mean ± SEM. *p < 0.05, **p < 0.01, ***p < 0.001; ns, no significance. *N* = 6–8 per group.

We then validated the effects of the IL-17-Glu axis on bone mass in vivo. Compared to the control group, treatment with IL-17 (50 µg/kg, i.p.) further decreased the trabecular bone in distal femur of OVX mice (Fig. 7A–E). Mice in the OVX + IL-17 group showed lower BV/TV, Tb.N and Tb.Th, but Tb.Sp were not significantly altered by IL-17 treatment (Fig. 7A–E). Despite these negative effects of IL-17 on bone mass, IL-17 did not aggravate trabecular bone loss in the V9302 (10 mg/kg) group, and no significant differences were observed in BV/TV, Tb.N, Tb.Th, and Tb.Sp between the V9302 (10 mg/kg) and the V9302 (10 mg/kg)+IL-17 group (Fig. 7A–E). However, supplementation of α-KG to the V9302 (10 mg/kg)+IL-17 group group inhibited the bone-increasing effects of V9302 (10 mg/kg), as revealed by decreased BV/TV and increased Tb.Sp in the IL-17 + V9302 (10 mg/kg)+α-KG group (Fig. 7A–E). HE and TRAP staining on femur slices indicated that IL-17 enhanced osteoclast formation and increased Oc.S/BS in the femur bone tissue of OVX mouse (Fig. 7F–I). However, V9302-induced osteoclast inhibition was not rescued by IL-17 mono-treatment (Fig. 7F–I), but was reversed by IL-17+α-KG cotreatment (Fig. 7F–I). These results suggest that IL-17 promotes osteoclast formation and accelerates bone loss in vivo, which requires the participation of Glu.

## Discussion

A previous study showed that Glu is essential for osteoclast differentiation, but the underlying mechanism remains un-explored [11]. In this study, we confirmed the role of Glu to drive osteoclast differentiation. Mechanistically, we found that Glu-deprivation reduces gene expression in the IL-17 signaling. More importantly, we revealed a novel role of IL-17 in regulating energy metabolism, which is Glu-dependent. IL-17 promotes osteoclast differentiation by increasing energy production, but this effect is inhibited by Glu-deprivation or by pharmacological block of Glu transport. In vivo treatment with IL-17 exacerbates OVX induced bone loss, which was suppressed by the Glu uptake inhibitor V9302. However, supplementation of the Glu metabolite α-KG partially rescued the effects of V9302 on bone mass. These results for the first time revealed that IL-17 can control energy metabolism by using Glu, and that targeting the IL-17–Glu axis by V9302 may be a potential strategy for the treatment of osteoclast-related diseases.

Glu is an essential substrate for cellular energy metabolism to meet the increasing demands of ATP production during cell growth and differentiation [20]. The amino acid transporter ASCT2 facilitates the transport of Glu into cells, which is then transformed into glutamate in the mitochondria through glutaminase-catalyzed deamidation [21]. Glutamate is then converted into α-KG that serves as an important intermediate in the TCA cycle [22]. Glu and its downstream metabolites are involved in the activity of macrophages. For example, one previous study revealed that Glu and α-KG can activate macrophages to participate in endotoxin-related immune response [23]. Another study found that Glu metabolism is controlled by the CD40 signal in macrophages to promote their anti-tumorigenic function [24]. During inflammation response, the production of many pro-inflammatory cytokines by macrophages, such as IL-1, IL-6, and TNF-α, requires Glu as energy sources [25]. These studies have revealed crucial roles of Glu in macrophage activation and function.

Osteoclasts also differentiate from macrophages in the bone marrow and consume a large amount of energy for differentiation and bone resorption [26]. RANKL stimulation showed a significant increase in OCR and ECAR, leading to increased ATP production, maximal respiration, glycolysis, and glycolytic capacity. This suggests an increase in mitochondrial respiration and glycolytic function during osteoclast differentiation. Glu is an important substrate for energy metabolism. A previous study reported that osteoclast differentiation is inhibited in Glu-free medium, but can be rescued by replenishment with the downstream product a-KG. In this study, we further proved that Glu and a-KG promoted ECAR, OCR, ATP production, maximal respiration, glycolysis, and glycolytic capacity, revealing a role of Glu and a-KG in osteoclast energy metabolism.

Combining transcriptome sequencing, we found that Glu deprivation does not affect MAPK and NFκB pathways, which are currently the most important pathway regulating osteoclast differentiation and function. Interestingly, we found for the first time that the IL-17 signaling pathway is also involved in Glu-regulated osteoclast differentiation. More importantly, we for the first time establish a link between the inflammatory IL-17 signal and energy metabolism. Besides, the KEGG results showed that The PI3K-Akt signaling pathway was also inhibited by Glu-deprivation. PI3K-Akt signaling pathway has a critical role in energy metabolism by regulating central regulator of aerobic glycolysis and autophagy [27]. The inhibiton of this pathway further revealed the metabolic influence of Glu in osteoclasts. However, we did not investigate whether the IL-17 and PI3K-Akt signaling pathway are interlinked in conditions of Glu deprivation, and whether the PI3K-Akt signaling pathway is involved in the IL-17-Glu axis remains to be explored. Current studies have shown that IL-17 is closely related to the regulation of bone metabolism. On the one hand, IL-17 can regulate the proliferation and differentiation of osteoblasts, promote the expression of RANKL in osteoblasts, and then indirectly promote osteoclast differentiation. On the other hand, IL-17 can also directly regulate the differentiation and function of osteoclasts. A large number of studies have shown that low concentration of IL-17 can promote osteoclast differentiation, while high concentration of IL-17 can inhibit osteoclast to a certain extent, but the specific mechanism needs further exploration. Our previous study revealed that IL-17 promotes osteoclast differentiation by activating the NF-κB and MAPK pathway [19]. In this study, we found genes in the IL-17 signaling pathway were downregulated by Glu deprivation. Treatment with IL-17 significantly increased energy metabolism and facilitated the formation of osteoclasts, while such effects were absent in BMDMs cultured in Glu-free medium. Interestingly, supplementing with α-KG could restore the promoting effect of IL-17 in the absence of Glu. In vivo experiments also showed that supplementing with IL-17 exacerbated OVX-induced bone loss, whereas inhibiting osteoclast uptake of Glu by V9302 suppressed the osteoclast promoting effect of IL-17. These results indicate that the role of IL-17 is tightly linked to Glu metabolism.

IL-17 is a proinflammatory cytokine produced mainly by TH_17_ cells. IL-17 is involved in chronic inflammation and drives a number of inflammation or autoimmune related diseases, including rheumatoid arthritis, psoriasis, Crohn’s disease, multiple sclerosis and asthma [28]. Monoclonal antibody products targeting IL-17 and/or IL-17R has have been approved for the treatment of immune-mediated diseases such as psoriasis, psoriatic arthritis, and ankylosing spondylitis [17]. The proinflammatory role of IL-17 is mainly mediated by activating many inflammatory pathways, such as the NFκB and MAPKs pathways. The activation of these pathways by IL-17 further induces the expression of various cytokines and chemokines [29]. However, there is currently no study to reveal the control of IL-17 on energy metabolism. Recent studies have shown that the production and secretion of IL-17 by mononuclear cells or T cells dependents on Glu [30, 31]. However, it remains unknown whether Glu mediates the biological or pathological function of IL-17. In this study, we for the first time found that IL-17 increases energy metabolism in BMDMs and promotes osteoclast differentiation, and this regulation is Glu dependent. We further revealed that blocking Glu transport can suppressed IL-17 induced osteoclast activation and bone loss, which may be a novel strategy to improve bone loss in patients with osteoporosis. Besides, as IL-17 is involved in a number of inflammation and autoimmune related disorders, the IL-17–Glu axis may be a promising target for the treatment of these diseases.

In conclusion, we revealed a previously unrecognized IL-17–Glu–energy metabolism axis. This regulation axis is important for osteoclast differentiation and contributes to osteoclast-mediated bone loss. Targeting the axis may be a novel therapeutic strategy for the treatment of osteoporosis and many other IL-17–related diseases.

### Supplementary information

Supplementary Material
