# Supplementary material for: IL-17 promotes osteoclast-induced bone loss by regulating glutamine-dependent energy metabolism
Source: Cell Death Dis. 2024 Feb 5;15(2):111. doi: 10.1038/s41419-024-06475-2 (PMC10844210; doi:10.1038/s41419-024-06475-2)

Supplementary Material

**Supplementary Table S1. Information on qRT-PCR primers used in this study**

| 编号 | PriRaer名称 | 序列(5'to3') |
| --- | --- | --- |
| 1 | **Actin (F)** | GGCTGTATTCCCCTCCATCG |
| 2 | **Actin (R)** | CCAGTTGGTAACAATGCCATGT |
| 3 | **Act1(F)** | TCCCGTGGAGGTTGATGAATC |
| 4 | **Act1(R)** | TCAGGGTGCCTTCTAAAGAAACT |
| 5 | **Traf6 (F)** | ACAGGCCATCCCAAGAATAGG |
| 6 | **Traf6 (R)** | AAGCCTCTGTTCATACCGTAGTA |
| 7 | **Cxcl10 (F)** | CCAAGTGCTGCCGTCATTTTC |
| 8 | **Cxcl10 (R)** | GGCTCGCAGGGATGATTTCAA |
| 9 | **Mmp3 (F)** | ACATGGAGACTTTGTCCCTTTTG |
| 10 | **Mmp3 (R)** | TTGGCTGAGTGGTAGAGTCCC |
| 11 | **Mmp9(F)** | CTGGACAGCCAGACACTAAAG |
| 12 | **Mmp9 (R)** | CTCGCGGCAAGTCTTCAGAG |
| 13 | **Mmp13 (F)** | CTTCTTCTTGTTGAGCTGGACTC |
| 14 | **Mmp13 (R)** | CTGTGGAGGTCACTGTAGACT |
| 15 | **Cebpb (F)** | GGCCCGGCTAGACAGTTAC |
| 16 | **Cebpb (R)** | GTTTCGGGACTTGATGCAAT |
| 17 | **Nfatc1**（F） | GGAGCGGAGAAACTTTGCG |
| 18 | **Nfatc1 (R)** | GTGACACTAGGGGACACATAACT |
| 19 | **MMP9 (F)** | CTGGACAGCCAGACACTAAAG |
| 20 | **MMP9 (R)** | CTCGCGGCAAGTCTTCAGAG |
| 21 | **CTSK (F)** | GAAGAAGACTCACCAGAAGCAG |
| 22 | **CTSK (R)** | TCCAGGTTATGGGCAGAGATT |
| 23 | **Trap(F)** | CACTCCCACCCTGAGATTTGT |
| 24 | **Trap(R)** | CATCGTCTGCACGGTTCTG |
| 25 | **Slc1a5(F)** | CATCAACGACTCTGTTGTAGACC |
| 26 | **Slc1a5(R)** | CGCTGGATACAGGATTGCGG |
| 27 | **GLS1(F)** | AGGGTCTGTTACCTAGCTTGG |
| 28 | **GLS1(R)** | ACGTTCGCAATCCTGTAGATTT |


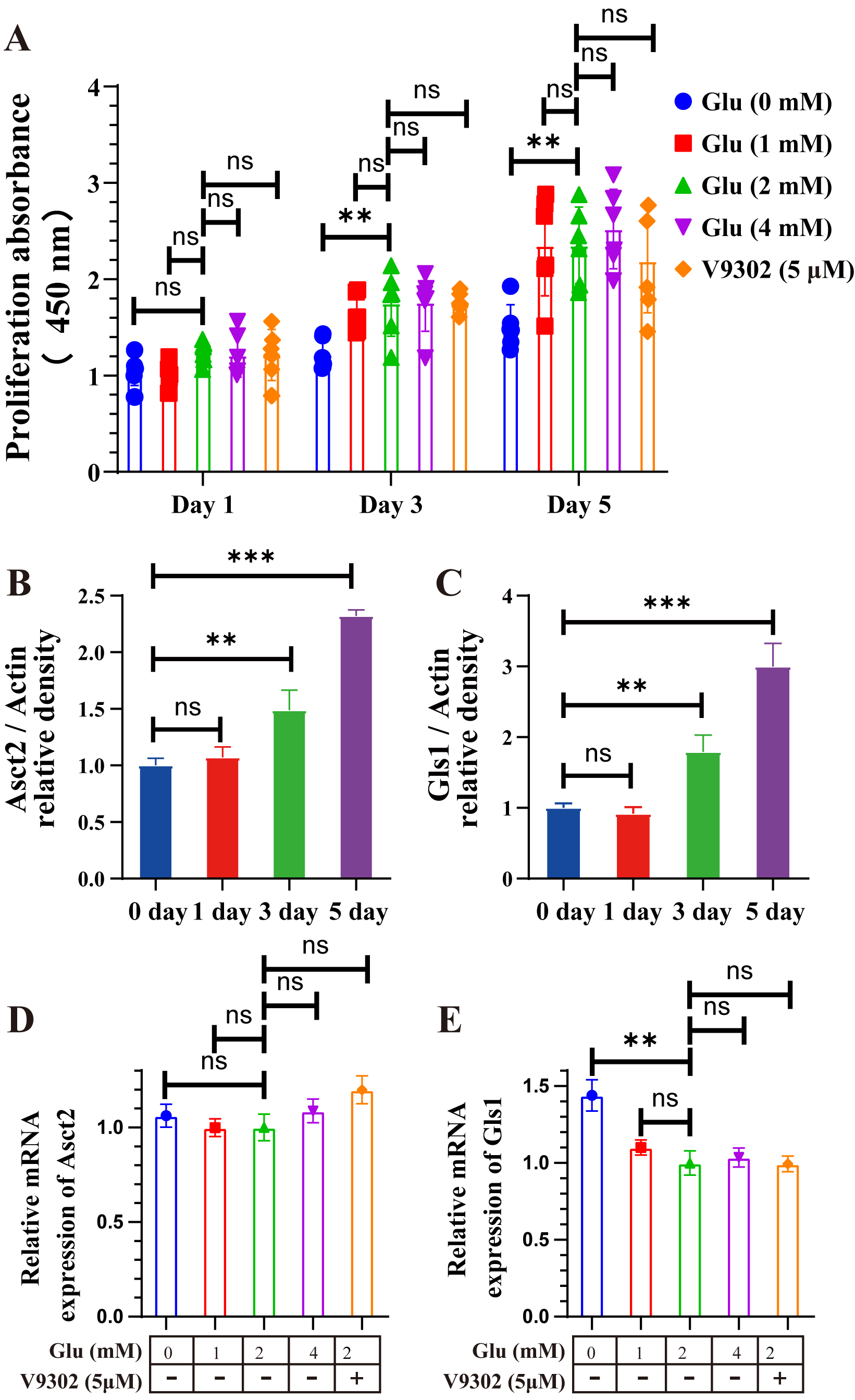


**Figure S1:** (A) Proliferation assays was conducted to clarify the impact of different concentration of Glu and Glu deprivation on BMDM. *p < 0.05, **p < 0.01, ***p < 0.001. ( B-C ) The expression of Asct2 and Gls1 at protein level on day 1, 3, and 5 during OC differentiation. Statistical significance: *p < 0.05, **p < 0.01, ***p < 0.001; ns, no significance. (D-E) Expression of Asct2 and Gls1 in different concentration of Glu medium and Glu deprived medium by qPCR test. Statistical significance: *p < 0.05, **p < 0.01, ***p < 0.001; ns, no significance.

**
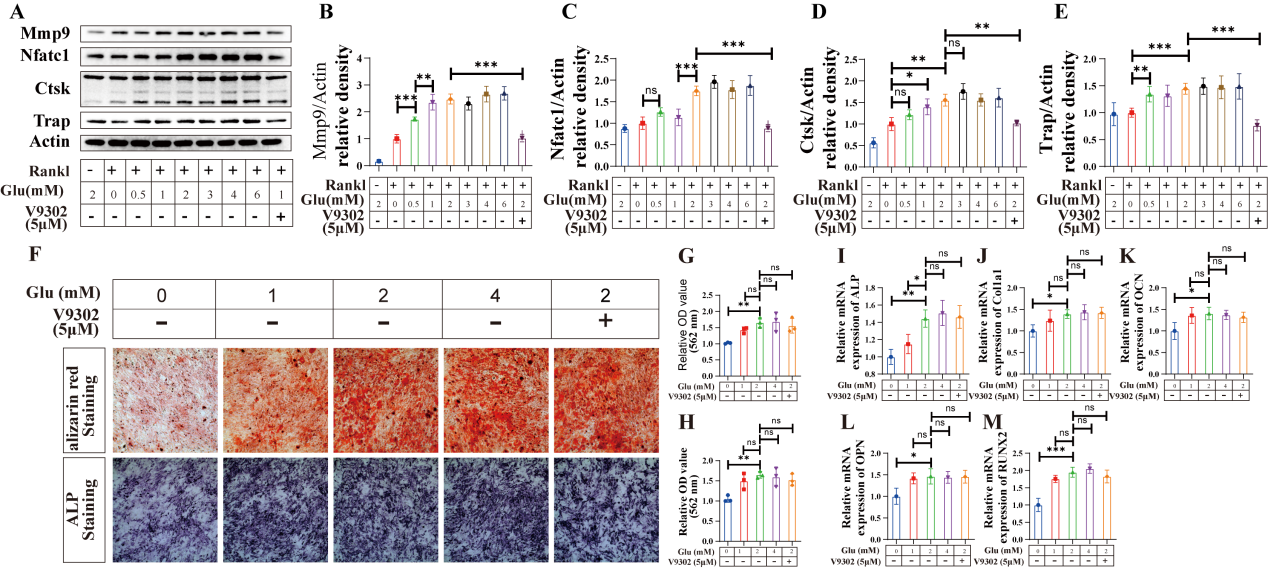
**

**Figure S2:** (A-E) BMDMs were cultured with the medium containing M-CSF, RANKL and various concentrations of Glu or were treated by 5 uM V9302 for 5 days. Protein expression levels of Nfatc1, Mmp9, Ctsk, and Acp5 were tested by Western blot assay. Statistical significance: *p < 0.05, **p < 0.01, ***p < 0.001; ns, no significance. (F-H) MC3T3-E1 cells were cultured in osteogenic induction medium with different concentrations of Glu. ALP staining and ARS staining were performed on days 7 and 21, respectively. Statistical significance: *p < 0.05, **p < 0.01, ***p < 0.001; ns, no significance. (I-M) MC3T3-E1 cells were treated with indicated concentrations of Glu under osteogenic induction, and the mRNA expression of osteoblast-specific genes were determined using qPCR on days 7. Statistical significance: *p < 0.05, **p < 0.01, ***p < 0.001; ns, no significance.


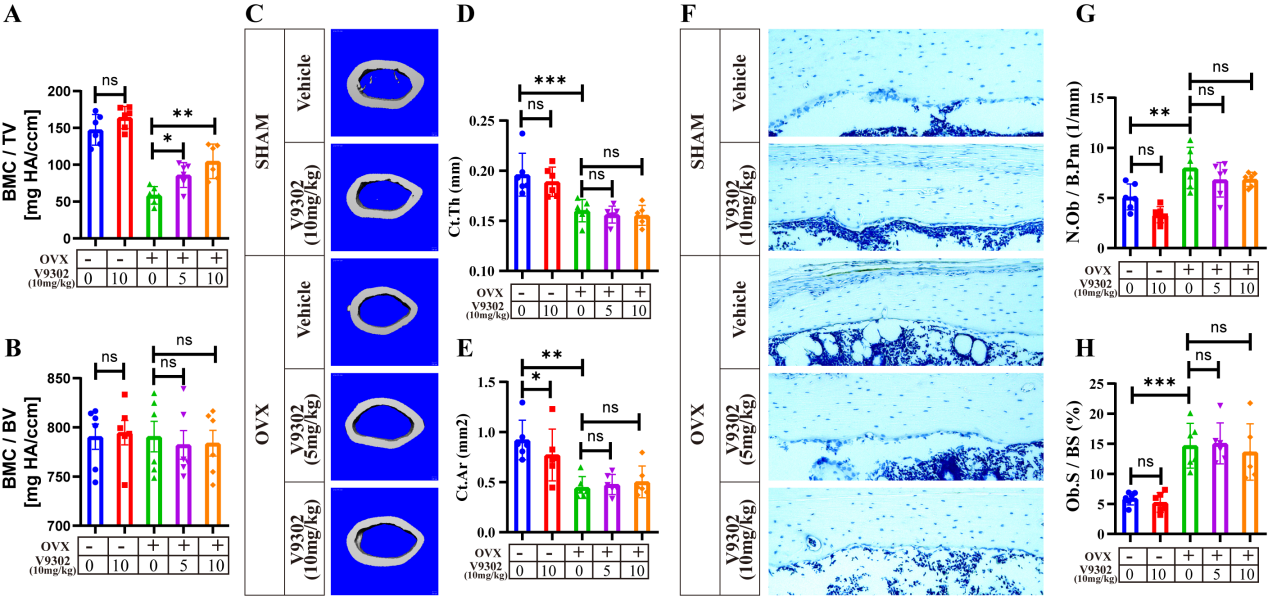


**Figure S3:** (A-B) (B-E) Quantitative analyses of bone structural parameters of the distal femurs, including Mean/Density of BV [mg HA/ccm] (BMC / BV)) and Mean/Density of TV [mg HA/ccm] (BMC / TV). (C-E) Representative three dimensional reconstructed μCT images of cortical bone from the femoral mid-shaft and quantitative analyses of cortical bone parameters, including Ct. Th and Ct. Ar. Statistical significance: *p < 0.05, **p < 0.01, ***p < 0.001; ns, no significance. (F) Representative toluidine blue staining showed the osteoblasts on endosteal surfaces. Quantification of osteoblast number per bone perimeter (N.Ob/B.Pm(1/mm)) (E) and osteoblast surface per bone perimeter (Ob.S/BS) (H) on endosteal surface. n = 6 per group. Statistical significance: *p < 0.05, **p < 0.01, ***p < 0.001; ns, no significance.


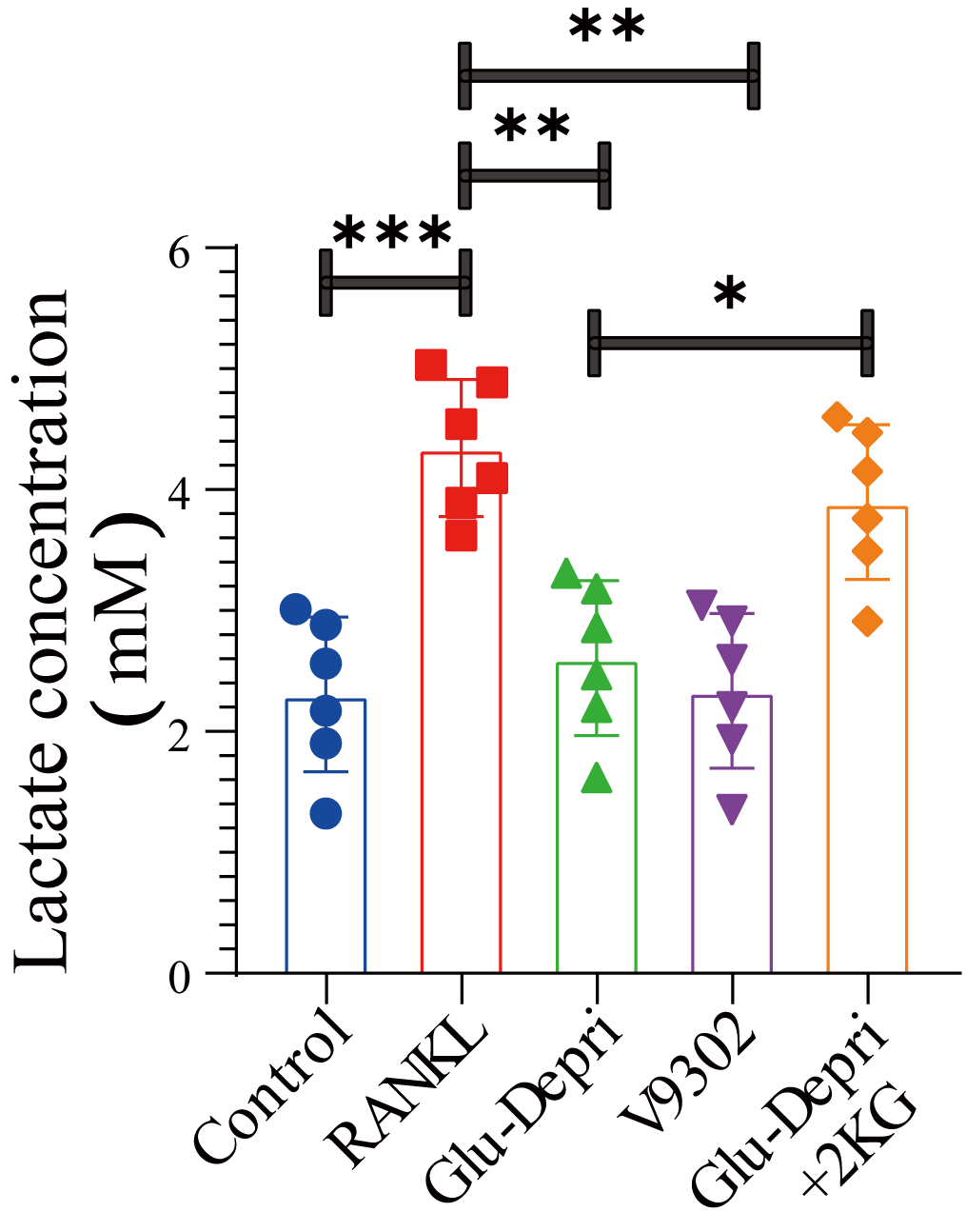


**Figure S4:** BMDMs were cultured with the Glu deprived medium that containing M-CSF ( 30ng/ml ) and RANKL (75 ng/mL) for 5 days, as well as treated with indicated concentration of α-KG. The levels of lactate were analyzed through Lactate Assay kit. *p < 0.05, **p < 0.01, ***p < 0.001.


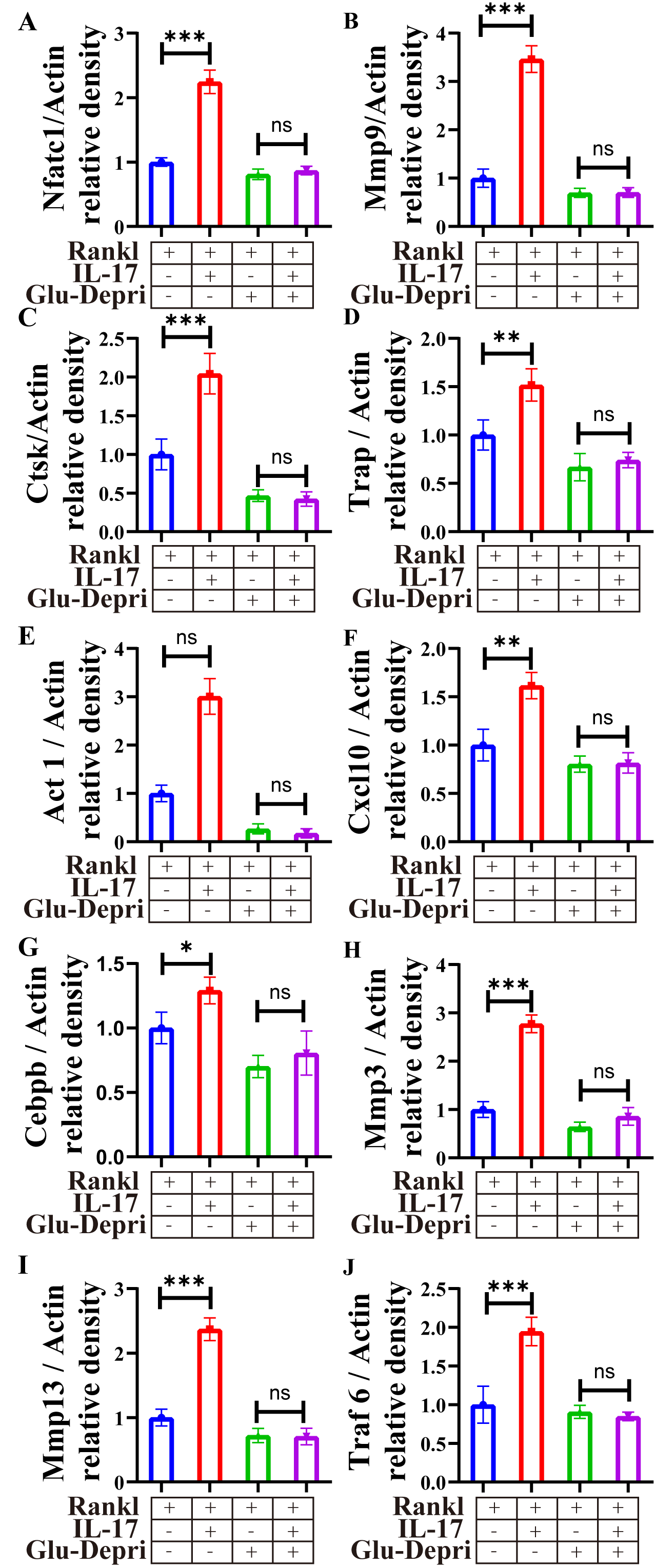


**Figure S5:** (A-J) BMDMs were seeded in 6 well plates and treated as Figure 5 described. Western blot assay examining the the expression of genes of osteoclast markers and genes in the IL-17 signaling pathway. *p < 0.05, **p < 0.01, ***p < 0.001; ns, no significance.

**Original data-qPCR and WB**

Figure 1A-B-qPCR

| Data Set | Target | Sample | Ctrl | Expression | Expression SEM | Corrected Expression SEM | Mean Cq | Cq SEM |
| --- | --- | --- | --- | --- | --- | --- | --- | --- |
| 1-SYBR | Actin | 0day |  |  |  |  | 15.17605 | 0.07429 |
| 1-SYBR | Actin | 1day |  |  |  |  | 15.41516 | 0.06226 |
| 1-SYBR | Actin | 3day |  |  |  |  | 14.46689 | 0.03204 |
| 1-SYBR | Actin | 5day |  |  |  |  | 15.35225 | 0.01192 |
| 1-SYBR | Asct2 | 0day |  | 0.42397 | 0.06415 | 0.06415 | 30.45986 | 0.06840 |
| 1-SYBR | Asct2 | 1day |  | 0.71893 | 0.07622 | 0.07622 | 30.46962 | 0.07080 |
| 1-SYBR | Asct2 | 3day |  | 0.89214 | 0.07603 | 0.07603 | 29.42761 | 0.05041 |
| 1-SYBR | Asct2 | 5day |  | 1.00000 | 0.05927 | 0.05927 | 30.26340 | 0.01082 |
| 1-SYBR | Gls1 | 0day |  | 1.00000 | 0.06070 | 0.06070 | 28.76029 | 0.02193 |
| 1-SYBR | Gls1 | 1day |  | 1.07189 | 0.27406 | 0.27406 | 28.96924 | 0.08842 |
| 1-SYBR | Gls1 | 3day |  | 1.89214 | 0.37532 | 0.37532 | 27.77417 | 0.04773 |
| 1-SYBR | Gls1 | 5day |  | 3.00000 | 0.25378 | 0.25378 | 28.45937 | 0.09662 |

Figure1J-M-qPCR

| Data Set | Target | Sample | Expression | Expression SEM | Corrected Expression SEM | Mean Cq | Cq SEM |
| --- | --- | --- | --- | --- | --- | --- | --- |
| 1-SYBR | Actin | 0 mM |  |  |  | 16.99280 | 0.08011 |
| 1-SYBR | Actin | 0.5 mM |  |  |  | 16.43089 | 0.04362 |
| 1-SYBR | Actin | 1.0 mM |  |  |  | 16.66230 | 0.07803 |
| 1-SYBR | Actin | 2.0 mM |  |  |  | 17.41992 | 0.10486 |
| 1-SYBR | Actin | 4.0 mM |  |  |  | 16.30354 | 0.07210 |
| 1-SYBR | Actin | V9302 |  |  |  | 15.94420 | 0.09153 |
| 1-SYBR | CTSK | 0 mM | 0.06321 | 0.00466 | 0.00466 | 25.92149 | 0.05041 |
| 1-SYBR | CTSK | 0.5 mM | 1.05653 | 0.13871 | 0.13871 | 24.16036 | 0.01082 |
| 1-SYBR | CTSK | 1.0 mM | 1.77865 | 0.28567 | 0.28567 | 24.14168 | 0.02193 |
| 1-SYBR | CTSK | 2.0 mM | 2.53296 | 0.33459 | 0.33459 | 24.74576 | 0.08842 |
| 1-SYBR | CTSK | 4.0 mM | 2.93611 | 0.23976 | 0.23976 | 23.56524 | 0.04773 |
| 1-SYBR | CTSK | V9302 | 0.36321 | 0.01195 | 0.01195 | 24.11352 | 0.09662 |
| 1-SYBR | NFATC1 | 0 mM | 0.69557 | 0.17029 | 0.17029 | 27.24732 | 0.07500 |
| 1-SYBR | NFATC1 | 0.5 mM | 0.79612 | 0.07395 | 0.07395 | 26.62677 | 0.05484 |
| 1-SYBR | NFATC1 | 1.0 mM | 0.95469 | 0.10527 | 0.10527 | 26.75916 | 0.09440 |
| 1-SYBR | NFATC1 | 2.0 mM | 1.25653 | 0.05352 | 0.05352 | 27.41761 | 0.05412 |
| 1-SYBR | NFATC1 | 4.0 mM | 1.32652 | 0.09294 | 0.09294 | 26.27769 | 0.05013 |
| 1-SYBR | NFATC1 | V9302 | 0.70162 | 0.07310 | 0.07310 | 26.19496 | 0.11395 |
| 1-SYBR | Mmp9 | 0 mM | 0.12269 | 0.02636 | 0.02636 | 19.65291 | 0.07958 |
| 1-SYBR | Mmp9 | 0.5 mM | 1.00653 | 0.14110 | 0.14110 | 18.17981 | 0.04496 |
| 1-SYBR | Mmp9 | 1.0 mM | 1.52743 | 0.11225 | 0.11225 | 18.22726 | 0.01417 |
| 1-SYBR | Mmp9 | 2.0 mM | 1.61576 | 0.22756 | 0.22756 | 18.96046 | 0.09745 |
| 1-SYBR | Mmp9 | 4.0 mM | 1.61039 | 0.12744 | 0.12744 | 17.84552 | 0.09248 |
| 1-SYBR | Mmp9 | V9302 | 0.18690 | 0.01898 | 0.01898 | 18.42151 | 0.05399 |
| 1-SYBR | Trap | 0 mM | 0.00772 | 0.00132 | 0.00132 | 20.81650 | 0.06025 |
| 1-SYBR | Trap | 0.5 mM | 0.46920 | 0.08367 | 0.08367 | 18.47085 | 0.07050 |
| 1-SYBR | Trap | 1.0 mM | 0.84177 | 0.17401 | 0.17401 | 18.44843 | 0.03915 |
| 1-SYBR | Trap | 2.0 mM | 1.05653 | 0.19084 | 0.19084 | 19.13124 | 0.09363 |
| 1-SYBR | Trap | 4.0 mM | 1.13776 | 0.10147 | 0.10147 | 17.95881 | 0.05978 |
| 1-SYBR | Trap | V9302 | 0.16120 | 0.01223 | 0.01223 | 18.44816 | 0.04396 |

Figure4G-L-qPCR

| Data Set | Target | Sample | Ctrl | Expression | Expression SEM | Corrected Expression SEM | Mean Cq | Cq SEM |
| --- | --- | --- | --- | --- | --- | --- | --- | --- |
| 1-SYBR | Actin | Control |  |  |  |  | 17.02391 | 0.02329 |
| 1-SYBR | Actin | Rankl |  |  |  |  | 16.74518 | 0.08417 |
| 1-SYBR | Actin | Rankl+Glu-depri |  |  |  |  | 17.11610 | 0.09426 |
| 1-SYBR | Act1 | Control |  | 1.00000 | 0.21190 | 0.21190 | 32.34276 | 0.08615 |
| 1-SYBR | Act1 | Rankl |  | 2.09119 | 0.38293 | 0.38293 | 31.74363 | 0.03227 |
| 1-SYBR | Act1 | Rankl+Glu-depri |  | 0.81393 | 0.10678 | 0.10678 | 32.52436 | 0.01598 |
| 1-SYBR | Cxcl10 | Control |  | 1.00000 | 0.30724 | 0.30724 | 30.11329 | 0.08631 |
| 1-SYBR | Cxcl10 | Rankl |  | 3.38165 | 0.40203 | 0.40203 | 29.30543 | 0.08450 |
| 1-SYBR | Cxcl10 | Rankl+Glu-depri |  | 0.95456 | 0.11583 | 0.11583 | 30.22568 | 0.05551 |
| 1-SYBR | Traf6 | Control |  | 1.00000 | 0.20210 | 0.20210 | 29.37806 | 0.09838 |
| 1-SYBR | Traf6 | Rankl |  | 1.92525 | 0.14009 | 0.14009 | 28.81484 | 0.08667 |
| 1-SYBR | Traf6 | Rankl+Glu-depri |  | 1.79438 | 0.28206 | 0.28206 | 29.21633 | 0.06722 |
| 1-SYBR | Cebp | Control |  | 1.00000 | 0.09887 | 0.09887 | 28.91686 | 0.01471 |
| 1-SYBR | Cebp | Rankl |  | 1.47339 | 0.14201 | 0.14201 | 28.46981 | 0.04991 |
| 1-SYBR | Cebp | Rankl+Glu-depri |  | 1.28802 | 0.28841 | 0.28841 | 28.89912 | 0.09221 |
| 1-SYBR | Mmp3 | Control |  | 1.00000 | 0.30719 | 0.30719 | 36.07450 | 0.01092 |
| 1-SYBR | Mmp3 | Rankl |  | 1.77755 | 0.24731 | 0.24731 | 35.54594 | 0.04738 |
| 1-SYBR | Mmp3 | Rankl+Glu-depri |  | 0.85500 | 0.18182 | 0.18182 | 36.23472 | 0.06450 |
| 1-SYBR | Mmp13 | Control |  | 1.00000 | 0.14112 | 0.14112 | 34.47480 | 0.05559 |
| 1-SYBR | Mmp13 | Rankl |  | 2.18829 | 0.45084 | 0.45084 | 33.85596 | 0.09923 |
| 1-SYBR | Mmp13 | Rankl+Glu-depri |  | 1.14432 | 0.18715 | 0.18715 | 34.50844 | 0.04465 |

Figure5G-qPCR

| Data Set | Target | Sample | Ctrl | Expression | Expression SEM | Corrected Expression SEM | Mean Cq | Cq SEM |
| --- | --- | --- | --- | --- | --- | --- | --- | --- |
| 1-SYBR | Actin | Control |  |  |  |  | 16.60399 | 0.03865 |
| 1-SYBR | Actin | IL17 |  |  |  |  | 16.22539 | 0.10053 |
| 1-SYBR | Actin | Glu-depri |  |  |  |  | 16.60901 | 0.06181 |
| 1-SYBR | Actin | Glu-depri+IL17 |  |  |  |  | 17.43695 | 0.10683 |
| 1-SYBR | NFATC1 | Control |  | 0.05099 | 0.21324 | 0.21324 | 28.57053 | 0.04532 |
| 1-SYBR | NFATC1 | IL17 |  | 0.09057 | 0.18742 | 0.18742 | 27.89387 | 0.03007 |
| 1-SYBR | NFATC1 | Glu-depri |  | 0.09426 | 0.07083 | 0.07083 | 28.78128 | 0.09989 |
| 1-SYBR | NFATC1 | Glu-depri+IL17 |  | 0.08234 | 0.12842 | 0.12842 | 29.59970 | 0.03994 |
| 1-SYBR | CTSK | Control |  | 1.00000 | 0.20729 | 0.20729 | 27.02398 | 0.05099 |
| 1-SYBR | CTSK | IL17 |  | 1.60203 | 0.08992 | 0.08992 | 26.44071 | 0.09057 |
| 1-SYBR | CTSK | Glu-depri |  | 0.50834 | 0.03674 | 0.03674 | 27.32284 | 0.09426 |
| 1-SYBR | CTSK | Glu-depri+IL17 |  | 0.47675 | 0.09923 | 0.09923 | 28.17865 | 0.08234 |
| 1-SYBR | MMP9 | Control |  | 1.00000 | 0.19528 | 0.19528 | 19.52818 | 0.01455 |
| 1-SYBR | MMP9 | IL17 |  | 3.31916 | 0.22087 | 0.22087 | 18.62855 | 0.02909 |
| 1-SYBR | MMP9 | Glu-depri |  | 0.42054 | 0.06771 | 0.06771 | 19.90939 | 0.04483 |
| 1-SYBR | MMP9 | Glu-depri+IL17 |  | 0.44822 | 0.06806 | 0.06806 | 20.70966 | 0.00419 |
| 1-SYBR | TRAP | Control |  | 1.00000 | 0.14824 | 0.14824 | 20.14448 | 0.07077 |
| 1-SYBR | TRAP | IL17 |  | 1.59600 | 0.26514 | 0.26514 | 19.56285 | 0.03463 |
| 1-SYBR | TRAP | Glu-depri |  | 0.59710 | 0.06026 | 0.06026 | 20.37345 | 0.07546 |
| 1-SYBR | TRAP | Glu-depri+IL17 |  | 0.58499 | 0.11469 | 0.11469 | 21.21030 | 0.04803 |

Figure5H-qPCR

| Data Set | Target | Sample | Ctrl | Expression | Expression SEM | Corrected Expression SEM | Mean Cq | Cq SEM |
| --- | --- | --- | --- | --- | --- | --- | --- | --- |
| 1-SYBR | Actin | Control |  |  |  |  | 17.43212 | 0.03314 |
| 1-SYBR | Actin | IL17 |  |  |  |  | 16.98320 | 0.09057 |
| 1-SYBR | Actin | Glu-depri |  |  |  |  | 16.79230 | 0.05718 |
| 1-SYBR | Actin | Glu-depri+IL17 |  |  |  |  | 17.49830 | 0.09732 |
| 1-SYBR | ACT1 | Control |  | 1.00000 | 0.17806 | 0.17806 | 33.77573 | 0.07308 |
| 1-SYBR | ACT1 | IL17 |  | 2.98631 | 0.21187 | 0.21187 | 32.85167 | 0.01241 |
| 1-SYBR | ACT1 | Glu-depri |  | 0.52269 | 0.20492 | 0.20492 | 33.41767 | 0.10049 |
| 1-SYBR | ACT1 | Glu-depri+IL17 |  | 0.52651 | 0.10254 | 0.10254 | 34.12050 | 0.10082 |
| 1-SYBR | CXCL10 | Control |  | 1.00000 | 0.12550 | 0.12550 | 32.82359 | 0.03011 |
| 1-SYBR | CXCL10 | IL17 |  | 2.22184 | 0.30155 | 0.30155 | 32.02796 | 0.06868 |
| 1-SYBR | CXCL10 | Glu-depri |  | 0.46332 | 0.07305 | 0.07305 | 32.51789 | 0.08465 |
| 1-SYBR | CXCL10 | Glu-depri+IL17 |  | 0.55372 | 0.19775 | 0.19775 | 33.14649 | 0.07792 |
| 1-SYBR | Traf6 | Control |  | 1.00000 | 0.24368 | 0.24368 | 29.35243 | 0.03193 |
| 1-SYBR | Traf6 | IL17 |  | 2.18714 | 0.32765 | 0.32765 | 28.56363 | 0.02894 |
| 1-SYBR | Traf6 | Glu-depri |  | 0.40761 | 0.16666 | 0.16666 | 29.10236 | 0.05778 |
| 1-SYBR | Traf6 | Glu-depri+IL17 |  | 0.50821 | 0.12105 | 0.12105 | 29.71257 | 0.06635 |
| 1-SYBR | Mmp3 | Control |  | 1.00000 | 0.11632 | 0.11632 | 35.26392 | 0.01525 |
| 1-SYBR | Mmp3 | IL17 |  | 2.37091 | 0.39136 | 0.39136 | 34.44008 | 0.04303 |
| 1-SYBR | Mmp3 | Glu-depri |  | 0.71647 | 0.10402 | 0.10402 | 34.76890 | 0.04870 |
| 1-SYBR | Mmp3 | Glu-depri+IL17 |  | 0.81052 | 0.11305 | 0.11305 | 35.42134 | 0.08265 |
| 1-SYBR | Mmp13 | Control |  | 1.00000 | 0.24578 | 0.24578 | 34.22539 | 0.09864 |
| 1-SYBR | Mmp13 | IL17 |  | 2.31888 | 0.33514 | 0.33514 | 33.41119 | 0.07109 |
| 1-SYBR | Mmp13 | Glu-depri |  | 0.43483 | 0.08292 | 0.08292 | 33.94725 | 0.04002 |
| 1-SYBR | Mmp13 | Glu-depri+IL17 |  | 0.51821 | 0.11201 | 0.11201 | 34.57706 | 0.02956 |
| 1-SYBR | cebp | Control |  | 1.00000 | 0.12194 | 0.12194 | 28.44147 | 0.07202 |
| 1-SYBR | cebp | IL17 |  | 1.47091 | 0.29249 | 0.29249 | 27.82497 | 0.06072 |
| 1-SYBR | cebp | Glu-depri |  | 0.74280 | 0.05426 | 0.05426 | 27.93079 | 0.06270 |
| 1-SYBR | cebp | Glu-depri+IL17 |  | 0.79277 | 0.11231 | 0.11231 | 28.60851 | 0.06668 |

Figure6H-qPCR

| Data Set | Target | Sample | Ctrl | Expression | Expression SEM | Corrected Expression SEM | Mean Cq | Cq SEM |
| --- | --- | --- | --- | --- | --- | --- | --- | --- |
| 1-SYBR | Actin | Rankl+glu-depri |  |  |  |  | 17.47044 | 0.09319 |
| 1-SYBR | Actin | Rankl+glu-depri+IL-17 |  |  |  |  | 16.46457 | 0.06613 |
| 1-SYBR | Actin | Rankl+glu-depri+IL-17+AKG |  |  |  |  | 17.29161 | 0.02713 |
| 1-SYBR | NFATC1 | Rankl+glu-depri |  | 1.00000 | 0.12713 | 0.12713 | 27.75238 | 0.04700 |
| 1-SYBR | NFATC1 | Rankl+glu-depri+IL-17 |  | 1.12071 | 0.27302 | 0.27302 | 26.29253 | 0.08727 |
| 1-SYBR | NFATC1 | Rankl+glu-depri+IL-17+AKG |  | 3.08865 | 0.64269 | 0.64269 | 26.86869 | 0.10245 |
| 1-SYBR | Mmp9 | Rankl+glu-depri |  | 1.00000 | 0.18557 | 0.18557 | 19.23499 | 0.01811 |
| 1-SYBR | Mmp9 | Rankl+glu-depri+IL-17 |  | 0.79358 | 0.10896 | 0.10896 | 17.92504 | 0.10577 |
| 1-SYBR | Mmp9 | Rankl+glu-depri+IL-17+AKG |  | 1.87788 | 0.28856 | 0.28856 | 18.56739 | 0.05156 |
| 1-SYBR | ctsk | Rankl+glu-depri |  | 1.00000 | 0.13023 | 0.13023 | 28.37085 | 0.09843 |
| 1-SYBR | ctsk | Rankl+glu-depri+IL-17 |  | 1.10144 | 0.12781 | 0.12781 | 26.91853 | 0.01196 |
| 1-SYBR | ctsk | Rankl+glu-depri+IL-17+AKG |  | 2.14702 | 0.22773 | 0.22773 | 27.64508 | 0.06538 |
| 1-SYBR | trap | Rankl+glu-depri |  | 1.00000 | 0.22797 | 0.22797 | 20.62079 | 0.04428 |
| 1-SYBR | trap | Rankl+glu-depri+IL-17 |  | 1.11614 | 0.10327 | 0.10327 | 19.16272 | 0.06917 |
| 1-SYBR | trap | Rankl+glu-depri+IL-17+AKG |  | 2.18880 | 0.15557 | 0.15557 | 19.88666 | 0.02270 |

Figure6I-qPCR

| Data Set | Target | Sample | Ctrl | Expression | Expression SEM | Corrected Expression SEM | Mean Cq | Cq SEM |
| --- | --- | --- | --- | --- | --- | --- | --- | --- |
| 1-SYBR | Actin | Rankl+glu-depri |  |  |  |  | 17.47044 | 0.09319 |
| 1-SYBR | Actin | Rankl+glu-depri+IL-17 |  |  |  |  | 16.46457 | 0.06613 |
| 1-SYBR | Actin | Rankl+glu-depri+IL-17+AKG |  |  |  |  | 17.29161 | 0.02713 |
| 1-SYBR | ACT1 | Rankl+glu-depri |  | 1.00000 | 0.11949 | 0.11949 | 32.02635 | 0.00788 |
| 1-SYBR | ACT1 | Rankl+glu-depri+IL-17 |  | 1.12071 | 0.27173 | 0.27173 | 30.97099 | 0.02602 |
| 1-SYBR | ACT1 | Rankl+glu-depri+IL-17+AKG |  | 3.08865 | 0.64484 | 0.64484 | 31.35775 | 0.03250 |
| 1-SYBR | CXCL10 | Rankl+glu-depri |  | 1.00000 | 0.16982 | 0.16982 | 31.58079 | 0.05327 |
| 1-SYBR | CXCL10 | Rankl+glu-depri+IL-17 |  | 1.37716 | 0.26205 | 0.26205 | 30.43594 | 0.04867 |
| 1-SYBR | CXCL10 | Rankl+glu-depri+IL-17+AKG |  | 3.14088 | 0.66940 | 0.66940 | 30.90491 | 0.03744 |
| 1-SYBR | Traf6 | Rankl+glu-depri |  | 1.00000 | 0.07568 | 0.07568 | 29.73766 | 0.03240 |
| 1-SYBR | Traf6 | Rankl+glu-depri+IL-17 |  | 1.09175 | 0.12015 | 0.12015 | 28.69366 | 0.05866 |
| 1-SYBR | Traf6 | Rankl+glu-depri+IL-17+AKG |  | 1.20282 | 0.08898 | 0.08898 | 29.47862 | 0.07492 |
| 1-SYBR | Mmp3 | Rankl+glu-depri |  | 1.00000 | 0.17898 | 0.17898 | 34.30171 | 0.04385 |
| 1-SYBR | Mmp3 | Rankl+glu-depri+IL-17 |  | 2.10587 | 0.52518 | 0.52518 | 32.97241 | 0.09209 |
| 1-SYBR | Mmp3 | Rankl+glu-depri+IL-17+AKG |  | 12.24650 | 2.15397 | 2.15397 | 33.03487 | 0.04243 |
| 1-SYBR | Mmp13 | Rankl+glu-depri |  | 1.00000 | 0.16765 | 0.16765 | 33.21892 | 0.09968 |
| 1-SYBR | Mmp13 | Rankl+glu-depri+IL-17 |  | 1.10396 | 0.07595 | 0.07595 | 32.17010 | 0.09815 |
| 1-SYBR | Mmp13 | Rankl+glu-depri+IL-17+AKG |  | 5.03999 | 0.14835 | 0.14835 | 32.33766 | 0.02340 |
| 1-SYBR | cebp | Rankl+glu-depri |  | 1.00000 | 0.15313 | 0.15313 | 27.36301 | 0.04767 |
| 1-SYBR | cebp | Rankl+glu-depri+IL-17 |  | 0.93416 | 0.05967 | 0.05967 | 26.38672 | 0.01563 |
| 1-SYBR | cebp | Rankl+glu-depri+IL-17+AKG |  | 2.61788 | 0.16558 | 0.16558 | 26.76623 | 0.10127 |

Figure S1-qPCR

| Data Set | Target | Sample | Ctrl | Expression | Expression SEM | Corrected Expression SEM | Mean Cq | Cq SEM |
| --- | --- | --- | --- | --- | --- | --- | --- | --- |
| 1-SYBR | Actin | 0 mM |  |  |  |  | 15.23247 | 0.09515 |
| 1-SYBR | Actin | 1 mM |  |  |  |  | 16.21767 | 0.08463 |
| 1-SYBR | Actin | 2 mM |  |  |  |  | 14.59282 | 0.06552 |
| 1-SYBR | Actin | 4 mM |  |  |  |  | 16.12395 | 0.08046 |
| 1-SYBR | Actin | V9302 |  |  |  |  | 15.66763 | 0.09192 |
| 1-SYBR | slc1a5 | 0 mM |  | 1.06240 | 0.11111 | 0.11111 | 32.32114 | 0.04435 |
| 1-SYBR | slc1a5 | 1 mM |  | 0.99893 | 0.08987 | 0.08987 | 33.33309 | 0.01698 |
| 1-SYBR | slc1a5 | 2 mM |  | 1.00000 | 0.12183 | 0.12183 | 31.70778 | 0.02090 |
| 1-SYBR | slc1a5 | 4 mM |  | 1.08768 | 0.10831 | 0.10831 | 33.20241 | 0.05037 |
| 1-SYBR | slc1a5 | V9302 |  | 1.19933 | 0.13579 | 0.13579 | 32.70365 | 0.02695 |
| 1-SYBR | gls1 | 0 mM |  | 1.43998 | 0.10191 | 0.10191 | 30.18762 | 0.02536 |
| 1-SYBR | gls1 | 1 mM |  | 1.10071 | 0.04962 | 0.04962 | 31.28950 | 0.10375 |
| 1-SYBR | gls1 | 2 mM |  | 1.00000 | 0.07866 | 0.07866 | 29.70633 | 0.09698 |
| 1-SYBR | gls1 | 4 mM |  | 1.03606 | 0.06191 | 0.06191 | 31.22207 | 0.05910 |
| 1-SYBR | gls1 | V9302 |  | 0.99374 | 0.05035 | 0.05035 | 30.78386 | 0.09439 |

Figure S2-qPCR

| Data Set | Target | Sample | Ctrl | Expression | Expression SEM | Corrected Expression SEM | Mean Cq | Cq SEM |
| --- | --- | --- | --- | --- | --- | --- | --- | --- |
| 1-SYBR | Actin | 0 mM |  |  |  |  | 16.02163 | 0.03650 |
| 1-SYBR | Actin | 1 mM |  |  |  |  | 15.86789 | 0.06125 |
| 1-SYBR | Actin | 2 mM |  |  |  |  | 14.64999 | 0.03614 |
| 1-SYBR | Actin | 4 mM |  |  |  |  | 15.85527 | 0.05050 |
| 1-SYBR | Actin | V9302 |  |  |  |  | 16.47155 | 0.02516 |
| 1-SYBR | Alp | 0 mM |  | 1.00000 | 0.09598 | 0.09598 | 30.28045 | 0.09420 |
| 1-SYBR | Alp | 1 mM |  | 1.14801 | 0.11296 | 0.11296 | 30.06676 | 0.05244 |
| 1-SYBR | Alp | 2 mM |  | 1.44273 | 0.10983 | 0.10983 | 28.74962 | 0.04516 |
| 1-SYBR | Alp | 4 mM |  | 1.50824 | 0.14968 | 0.14968 | 29.93561 | 0.09443 |
| 1-SYBR | Alp | V9302 |  | 1.46177 | 0.13207 | 0.13207 | 30.56549 | 0.05829 |
| 1-SYBR | col1a2 | 0 mM |  | 1.00000 | 0.14144 | 0.14144 | 12.39819 | 0.04364 |
| 1-SYBR | col1a2 | 1 mM |  | 1.23878 | 0.24436 | 0.24436 | 12.15145 | 0.00621 |
| 1-SYBR | col1a2 | 2 mM |  | 1.39031 | 0.10346 | 0.10346 | 10.88344 | 0.07754 |
| 1-SYBR | col1a2 | 4 mM |  | 1.43634 | 0.17928 | 0.17928 | 12.07457 | 0.01941 |
| 1-SYBR | col1a2 | V9302 |  | 1.41948 | 0.13350 | 0.13350 | 12.69598 | 0.08485 |
| 1-SYBR | opn | 0 mM |  | 1.00000 | 0.19564 | 0.19564 | 13.48324 | 0.02443 |
| 1-SYBR | opn | 1 mM |  | 1.41613 | 0.12812 | 0.12812 | 13.17840 | 0.05081 |
| 1-SYBR | opn | 2 mM |  | 1.46359 | 0.17791 | 0.17791 | 11.94618 | 0.03574 |
| 1-SYBR | opn | 4 mM |  | 1.43987 | 0.13918 | 0.13918 | 13.15855 | 0.07615 |
| 1-SYBR | opn | V9302 |  | 1.45705 | 0.15526 | 0.15526 | 13.76969 | 0.09826 |
| 1-SYBR | ocn | 0 mM |  | 1.00000 | 0.19685 | 0.19685 | 36.64819 | 0.03924 |
| 1-SYBR | ocn | 1 mM |  | 1.35959 | 0.18589 | 0.18589 | 36.36105 | 0.04652 |
| 1-SYBR | ocn | 2 mM |  | 1.40387 | 0.14566 | 0.14566 | 35.12923 | 0.02601 |
| 1-SYBR | ocn | 4 mM |  | 1.36982 | 0.11245 | 0.11245 | 36.34516 | 0.09125 |
| 1-SYBR | ocn | V9302 |  | 1.31624 | 0.12618 | 0.12618 | 36.97878 | 0.04663 |
| 1-SYBR | runx2 | 0 mM |  | 1.00000 | 0.14592 | 0.14592 | 28.85987 | 0.08230 |
| 1-SYBR | runx2 | 1 mM |  | 1.23878 | 0.24811 | 0.24811 | 28.61313 | 0.06878 |
| 1-SYBR | runx2 | 2 mM |  | 1.39031 | 0.10961 | 0.10961 | 27.34512 | 0.03331 |
| 1-SYBR | runx2 | 4 mM |  | 1.43634 | 0.17576 | 0.17576 | 28.53625 | 0.00875 |
| 1-SYBR | runx2 | V9302 |  | 1.41948 | 0.13446 | 0.13446 | 29.15766 | 0.01264 |


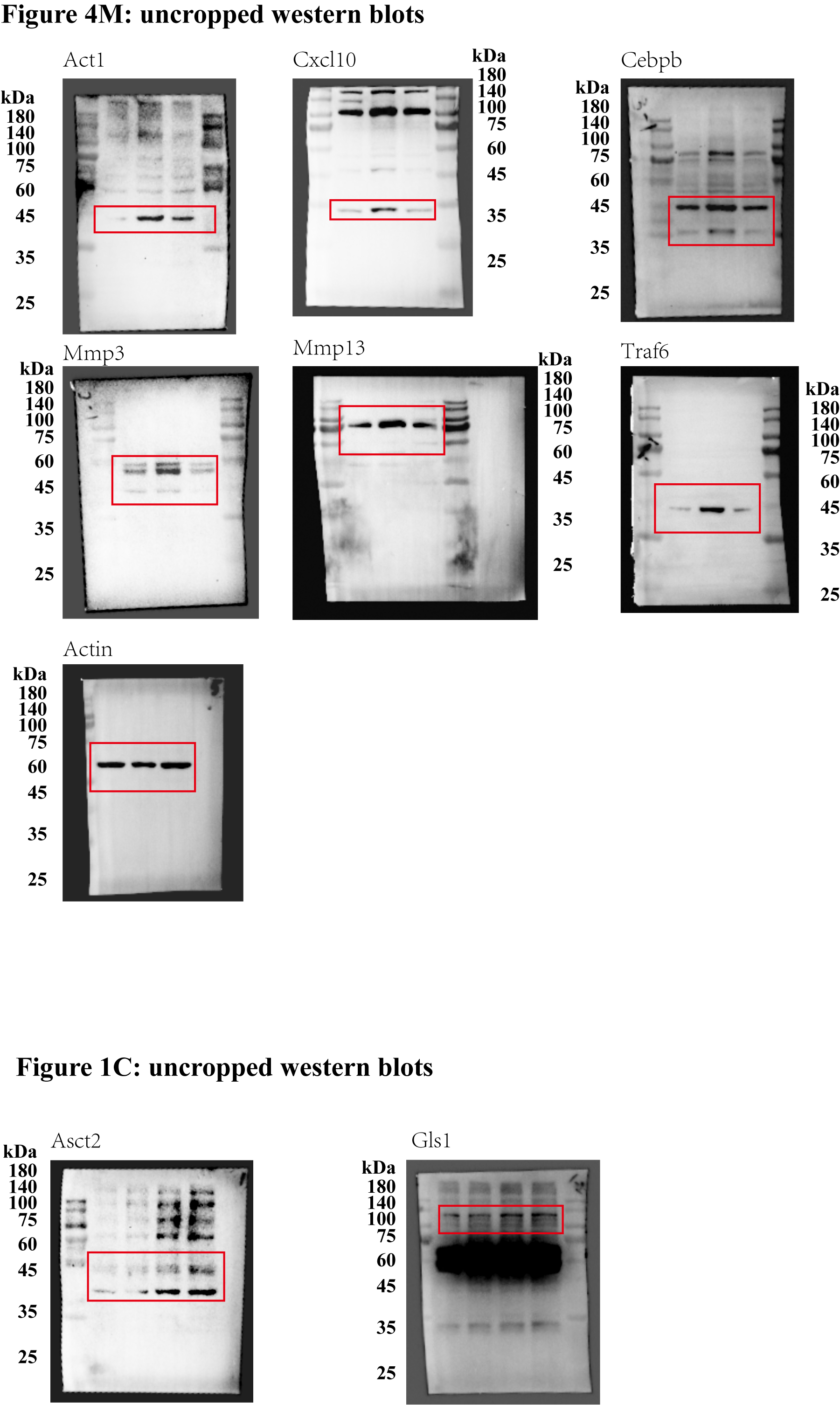


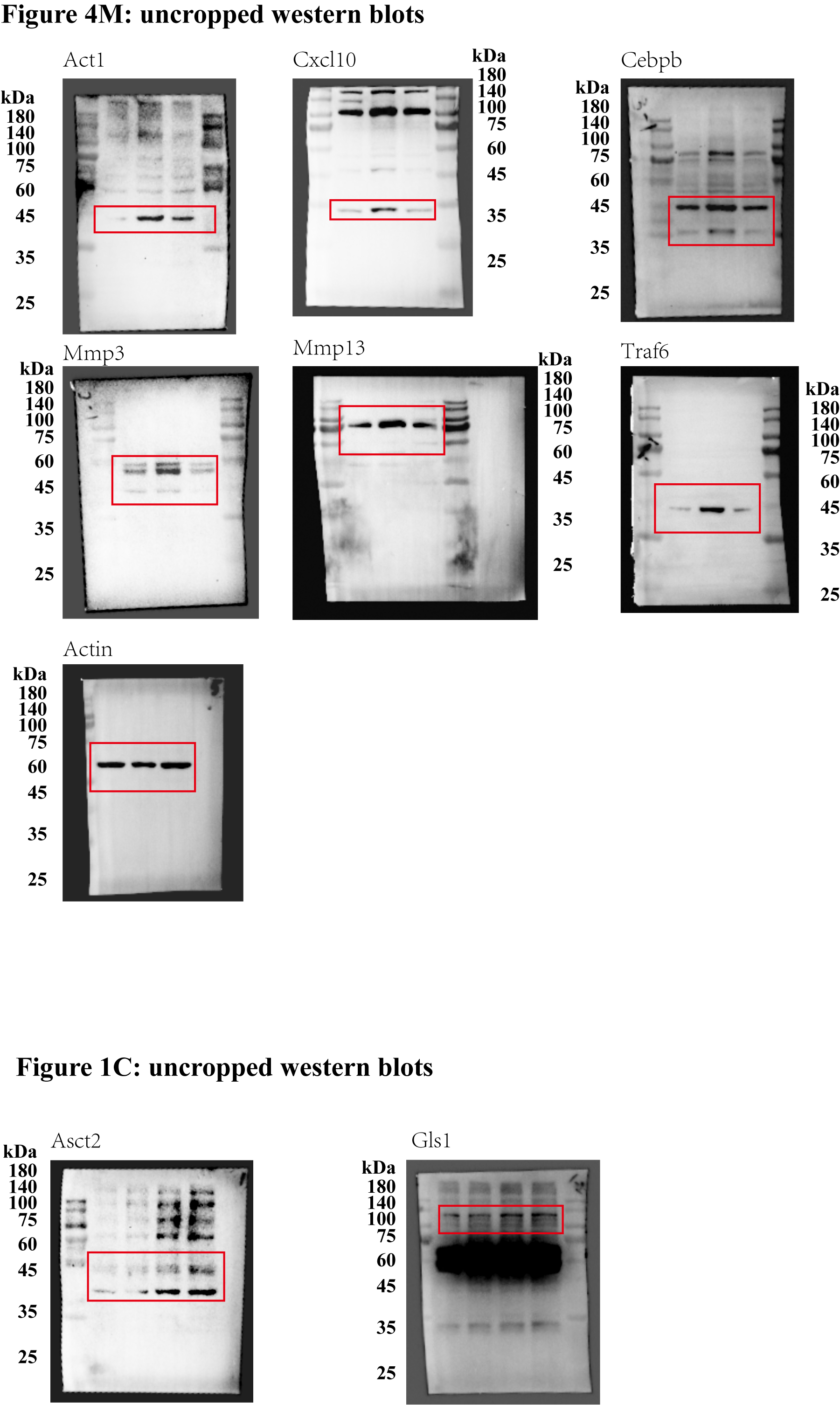


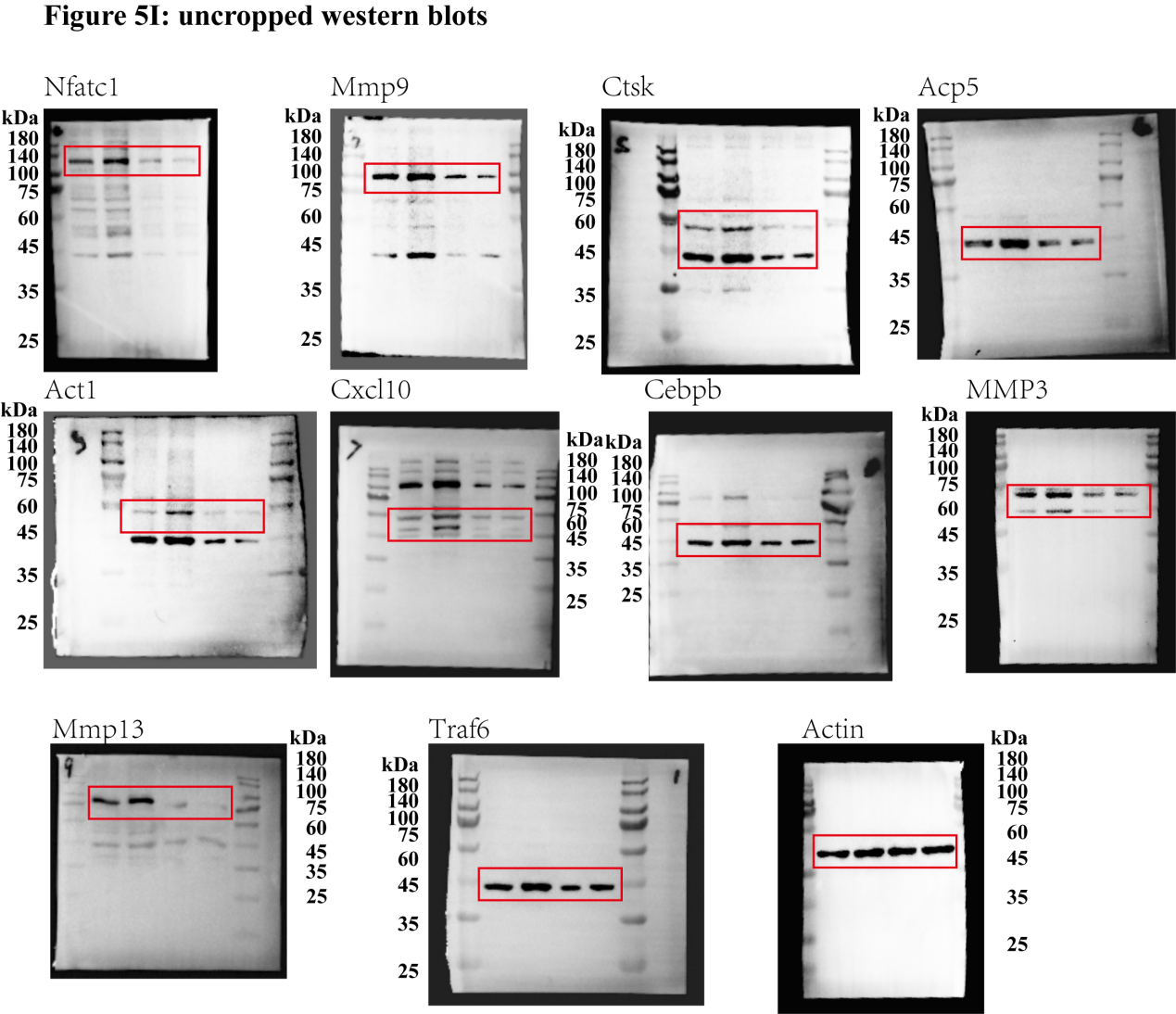
**Figure S2-wb**


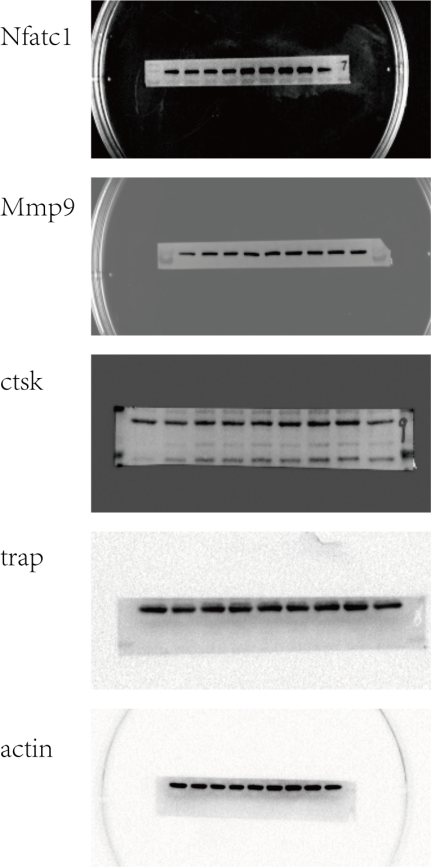

Supplement: Supplementary file 1 — Supplementary Material [file 41419_2024_6475_MOESM1_ESM.docx]
